# SWI/SNF complexes are required for retinal pigmented epithelium differentiation and for the inhibition of cell proliferation and neural differentiation programs

**DOI:** 10.1242/dev.201488

**Published:** 2023-08-21

**Authors:** Shai Ovadia, Guizhong Cui, Ran Elkon, Mazal Cohen-Gulkar, Nitay Zuk-Bar, Tran Tuoc, Naihe Jing, Ruth Ashery-Padan

**Affiliations:** ^1^ Department of Human Molecular Genetics and Biochemistry, Faculty of Medicine and Sagol School of Neuroscience, Tel Aviv University, Tel Aviv 69978, Israel; ^2^ Guangzhou National Laboratory, Department of Basic Research, Guangzhou 510005, China; ^3^ Department of Human Genetics, Ruhr University of Bochum, 44791 Bochum, Germany

**Keywords:** RPE, SWI/SNF, Gene regulatory networks, Eye development, Mouse

## Abstract

During embryonic development, tissue-specific transcription factors and chromatin remodelers function together to ensure gradual, coordinated differentiation of multiple lineages. Here, we define this regulatory interplay in the developing retinal pigmented epithelium (RPE), a neuroectodermal lineage essential for the development, function and maintenance of the adjacent retina. We present a high-resolution spatial transcriptomic atlas of the developing mouse RPE and the adjacent ocular mesenchyme obtained by geographical position sequencing (Geo-seq) of a single developmental stage of the eye that encompasses young and more mature ocular progenitors. These transcriptomic data, available online, reveal the key transcription factors and their gene regulatory networks during RPE and ocular mesenchyme differentiation. Moreover, conditional inactivation followed by Geo-seq revealed that this differentiation program is dependent on the activity of SWI/SNF complexes, shown here to control the expression and activity of RPE transcription factors and, at the same time, inhibit neural progenitor and cell proliferation genes. The findings reveal the roles of the SWI/SNF complexes in controlling the intersection between RPE and neural cell fates and the coupling of cell-cycle exit and differentiation.

## Teaser

**Summary:** Spatial transcriptomic of embryonic control and SWI/SNF-deficient RPE uncovers RPE-specific gene regulatory networks and their dependence on the SWI/SNF complexes, which further inhibit the neural and cell proliferation genes.

## INTRODUCTION

The cell-specific transcriptome is acquired gradually during embryogenesis through a sequential, multistep process that is spatially and temporally regulated. We study how these transcriptional transitions are regulated by transcription factors (TFs) and chromatin remodelers in the differentiation of the retinal pigmented epithelium (RPE), an essential tissue for eye organogenesis and for retinal function throughout life (Strauss, 2005). The RPE is also an excellent model for studying the transition of neural progenitors to highly specialized tissue of the central nervous system. The RPE originates from neural progenitors of the anterior neural tube that form the optic vesicles, which later give rise to the optic cups. The optic cups contain an outer layer of pigmented epithelium (PE) progenitors and an inner layer of retinal progenitor cells (RPCs). Most of the PE progenitors differentiate to form the RPE, whereas those located in the distal tip of the optic cup form the pigmented layers of the ciliary body and iris (Davis-Silberman and Ashery-Padan, 2008).

Maturation of the PE to RPE occurs gradually, with the appearance of pigmentation, acquisition of polarity, adhesion, and barrier formation occurring during the earlier developmental stages, and the expression of visual cycle genes and phagocytosis activity being acquired later in development, in conjunction with photoreceptor differentiation (Fields et al., 2020). RPE maturation occurs in a defined spatial pattern in the optic cup, from central-dorsal towards distal-ventral regions. Thus, the location of the progenitor in the optic cup corresponds to the stage of tissue maturation. Another important feature of the RPE is its potential to dedifferentiate/transdifferentiate into neuroretinal tissue in response to external or genetic perturbations (Fuhrmann et al., 2014). Currently, the mechanism underlying the dedifferentiation/transdifferentiation properties of the embryonic RPE and how this plasticity is restricted during tissue maturation remain mostly unknown.

Specification and differentiation of the RPE depend on external cues from the surrounding ocular mesenchyme (Me) and surface ectoderm (Cardozo et al., 2020; Fuhrmann et al., 2014). Genetic studies and tissue transplantation in animal models have established roles for Wnt/β-catenin, BMPs and Hippo signaling in RPE cell fate and maturation (Carpenter et al., 2015; Kim et al., 2016; Martinez-Morales et al., 2003; Miesfeld et al., 2015; Steinfeld et al., 2013; Westenskow et al., 2009, 2010). External cues impact the expression and activity of the RPE TFs, including microphthalmia-associated transcription factor (MITF), which is important for the differentiation of melanocytes and ocular pigment cells (Ma et al., 2019; Masuda et al., 2014; Scholtz and Chan, 1987). Functioning upstream and together with MITF is the paired-type homeodomain TF Drosophila orthodenticle homolog 2 (OTX2). *Otx2* is essential in the early neuroectoderm for formation of the forebrain and midbrain and, subsequently, for specification of the eye field, which forms the optic vesicles (Acampora et al., 1995; Ang et al., 1996; Martinez-Morales et al., 2001; Matsuo et al., 1995; Zuber et al., 2003). *Otx2* continues to be expressed in the RPE throughout life and is required for the expression of many RPE genes (Beby et al., 2010; Housset et al., 2013; Masuda and Esumi, 2010). All of these TFs function in the developing RPE from the optic vesicle stage, yet the maturation process must depend on temporally expressed factors. In this regard, the TFs PAX6 and SOX9 have been documented to control the early and late stages, respectively, of RPE differentiation; *Pax6* is expressed transiently in the RPE progenitors, controlling the level and activity of *Mitf* and inhibiting the expression of *Sox9*, which in turn is expressed later in development and is required for the expression of RPE maturation genes, such as *Rpe65*, *Vmd2* (*Best1*), *Angptl4* and *Vegf* (Cohen-Tayar et al., 2018; Raviv et al., 2014). Despite the identification of the aforementioned regulators, our understanding of the gene regulatory networks governing the transition from PE to RPE remains limited.

Essential regulators of TF activity are the chromatin modifiers and remodelers (Zaret and Carroll, 2011). Among these, the highly studied ATP-dependent SWI/SNF [SMARCA4 (BRG1)/SMARCA2 (Brm)-associated factor (BAF)] chromatin remodeling complexes have been shown to promote chromatin accessibility by recruiting histone modifiers and evicting the Polycomb repressive complexes (Ho et al., 2011; Kadoch et al., 2017; Phelan et al., 1999). These SWI/SNF complexes are thought to play a crucial role in establishing the chromatin landscape that enables the regulatory activity of tissue-specific TFs (Hoffman et al., 2018). The tissue-specific activity of the SWI/SNF complexes is achieved through combinatorial assembly of their multiple subunits and direct interaction with lineage-specific TFs, including those that are important for RPE differentiation [e.g. PAX6 (Narayanan et al., 2018; Ninkovic et al., 2013; Tuoc et al., 2013a), MITF (Laurette et al., 2015) and OTX2 (Cohen-Gulkar et al., 2023)]. Nevertheless, the roles of these complexes in RPE differentiation remain unexplored.

In this study, we combined PE-specific conditional inactivation of the SWI/SNF complexes with geographical position sequencing (Geo-seq; Chen et al., 2017) to generate a comprehensive transcriptomic atlas from which we could infer the gene regulatory networks governing the complex differentiation program of the RPE. We further show, through PE-specific conditional inactivation, that the SWI/SNF complexes regulate the expression and activity of key TFs controlling the RPE differentiation program while functioning to repress cell proliferation and neural gene expression in the PE. The findings place the SWI/SNF complexes upstream of two crucial developmental crossroads: the decision between neural and RPE cell fates, and that between cell proliferation and cell differentiation.

## RESULTS

### Determining the spatial transcriptome of the developing murine RPE during normal development and following SWI/SNF inactivation

**Fig. 1.**
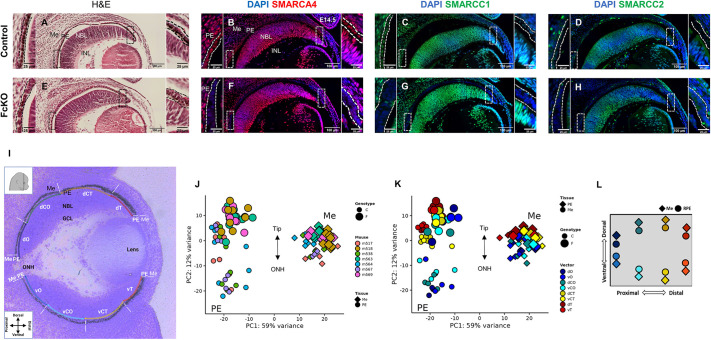
**Conditional inactivation of the SWI/SNF complex in developing RPE and Geo-seq analyses.** (A-H) Sections of control (A-D) and *Smarcc1^loxP/loxP^;Smarcc2^loxP/loxP^;DCT-Cre* (FcKO; E-H) E14.5 eyes analyzed by histology (Hematoxylin and Eosin, A,E) and antibody labeling for detection of SMARCA4 (B,F), SMARCC1 (C,G) and SMARCC2 (D,H). Insets show magnifications of the boxed areas. Scale bars: 100 μm in the low magnification images; 25 μm in the high magnification images. DAPI was used as counterstain. Dashed lines delineate the PE. (I) Position of the PE and Me samples from the E14.5 optic cups used for Geo-seq; dorsal (d) and ventral (v) samples were collected along proximal (O, close to optic nerve head) to distal (T, tip of the optic cup, close to lens) axes of the optic cup. The central (C) positions are CO and CT, respectively (color code corresponds to position). (J,K) PCA plots of the normalized expression data generated by DESeq2, highlighted by color code for each embryo (J) or position in the optic cup (K); symbol shape represents the tissue (PE or Me), and symbol size represents the genotype. (L) Football plot representation of the transcriptomic data used to visualize the expression of any gene of interest in control or FcKO samples. GCL, ganglion cell layer; INL, inner nuclear layer; NBL, neuroblast cell layer; Me, ocular mesenchyme; ONH, optic nerve head; PE, pigmented epithelium.

To obtain a high-resolution transcriptomic profile of the developing RPE and to determine the role of SWI/SNF in RPE maturation, we focused on embryonic day (E) 14.5, a transitory stage in eye development, when the maturation of PE into RPE is ongoing. At this stage, a single layer of PE surrounds the developing retina (Fig. 1A), and it is molecularly heterogeneous based on opposing expression gradients of the early and late RPE TFs PAX6 and SOX9, respectively (Cohen-Tayar et al., 2018). At this stage, both the catalytic subunit SMARCA4 (also known as BRG1) and the structural subunit SMARCC1 (also known as BAF155) were detected by antibody labeling in numerous optic cup cell types: PE, RPCs, ganglion cell precursors in the inner nuclear layer and in the surrounding Me (Fig. 1B,C, Fig. S1A,B). In accordance with its role in tissue maturation (Tuoc et al., 2017, 2013a), SMARCC2 (also known as BAF170) expression was higher in the inner nuclear layer, populated by ganglion and early-born amacrine precursors, compared with low expression in the RPCs and PE (Fig. 1D, Fig. S1A).

We generated PE-specific deletion of both *Smarcc1* and *Smarcc2* using the respective conditional alleles and the dopachrome tautomerase (*Dct*, also known as *Tyrp2*) driver *DCT-Cre*, which is active in the pigmented progenitors of the optic cup (Choi et al., 2012; Davis et al., 2009; Tuoc et al., 2013b). Loss from the PE of the two subunits [*Smarcc1^loxP/loxP^;Smarcc2^loxP/loxP^;DCT-Cre*; termed full conditional knockout (FcKO) embryos] resulted in hypopigmentation and thickening of the PE layer based on Hematoxylin and Eosin analysis (Fig. 1E). In the FcKO PE, the three subunits (SMARCC1, SMARCC2 and SMARCA4) were below detection limits, but were maintained in the adjacent ocular cell types, corresponding to the pattern of activity of *DCT-Cre* (Fig. 1F-H, Fig. S1). The marked reduction of SMARCA4 protein in the FcKO PE, although the *Smarca4* gene was not deleted, corresponds with a previous report showing proteasomal degradation of the complex in the absence of both SMARCC1 and SMARCC2 (Narayanan et al., 2018).

To determine the transcriptome of a low number of PE cells and adjacent ocular Me in both the control and FcKO embryos, we implemented Geo-seq (Chen et al., 2017; Peng et al., 2020) on four control and three FcKO eyes, each from a different embryo, enabling a statistical evaluation of the variations between and within the samples. Using laser-capture microdissection (LCM), we isolated eight adjacent samples along the proximal-distal axis from both dorsal and ventral positions of the PE and the adjacent Me (Fig. 1I; see Materials and Methods). The samples were named according to their respective genotype (control or FcKO), tissue of origin (PE or Me) and spatial position along the dorsal-ventral and proximal-distal axes (color coded in Fig. 1I, Table S1). We used the Smart-Seq2 protocol and obtained a mean depth of 30 million sequenced reads per sample. The raw reads were mapped onto the mouse reference genome (mm10) and an average of about 18,000 unique genes were identified per sample with a mapping ratio of 65-90% (average of 77%; Fig. S2A). Principal component analysis (PCA) and pairwise correlation followed by hierarchal clustering showed a high correlation between the samples isolated from the same regions and genotypes from different embryos (Fig. 1J,K, Fig. S2B).

This analysis provided a comprehensive catalog of the transcripts in defined locations along the proximal-distal and dorsal-ventral axes of the PE and Me optic cup lineages in controls and following SWI/SNF inactivation in the PE. The expression profile of each gene of interest was graphically represented by a 2D football plot (Fig. 1L). Football plots illustrating the relative expression (Z-scores) in the control of genes known to be expressed in the RPE (*Mitf*, *Sox9* and *Pax6*; Cohen-Tayar et al., 2018; Fuhrmann et al., 2014) and Me (*Pitx2*, *Dkk2* and *Col3a1*; Evans and Gage, 2005; Gage et al., 2008) demonstrated the data's faithful representation of tissue-specific gene-expression profiles (Fig. S2C). The football plot representations of the spatial optic cup transcriptomics (SPOT) data are available online (https://shaiovadia.shinyapps.io/NewShiny_Publication/).

**Fig. 2.**
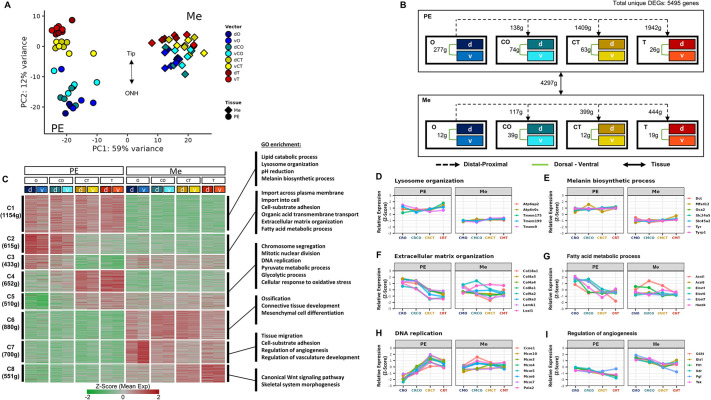
**Positional transcriptomic atlas of the developing pigmented epithelium (PE) and ocular mesenchyme (Me) progenitors.** (A) PCA plot of the control samples (color indicates spatial position, shape indicates tissue). (B) Scheme describing the statistical comparisons analyzed by DESeq2 between and within PE and Me samples. The number of differentially expressed genes (DEGs, [|log2(fold change)|>0.5, *P-*adj<0.05]; g, genes) for each comparison is indicated. (C) Heat map (K-means, K=8) representing the clustering analysis results based on the average positional expression of all DEGs. On the right are selected enriched GO terms for genes with similar expression patterns. (D-I) Line plots showing the proximal-distal expression patterns of selected genes from the enriched GO terms in both PE and Me. Positions along the proximal (O, position close to optic nerve head) to distal (T, tip of the optic cup, close to lens) axes of the optic cup are indicated. The central positions are termed CO and CT (see Fig. 1I).

To determine the transcriptional networks of RPE differentiation, we focused on the control samples. PCA plots of the control samples showed that the major variance factor (PC1) is the tissue of origin (PE or Me). The second variance factor (PC2) was attributed to the positions of the PE and Me samples along the proximal-distal axis (Fig. 2A). We determined the differentially expressed genes (DEGs) between the PE and Me using DESeq2 (Love et al., 2014). This analysis identified a total of 4297 DEGs, with 2184 genes more highly expressed in the PE and 2113 more highly expressed in the Me [|log2(fold change)|>0.5, *P-*adj<0.05] (Fig. 2B, Fig. S2D). Corresponding with the biological activities of PE and Me, gene set enrichment analysis (GSEA) detected enrichment in lysosomal and pigment-biosynthesis processes for the PE, and vasculature development and ossification for the Me (Fig. S2E,F).

To identify DEGs along the proximal-distal axis within the PE or Me, we compared, for each tissue, the expression in the most proximal position (termed O) to all of the other positions (termed CO, CT, and T; Fig. 2B). To identify the differences along the dorsal-ventral axis, we further determined, for each tissue (PE or Me), the DEGs between the dorsal (d) ventral (v) samples (dO versus vO, dCO versus vCO, dCT versus vCT, and dT versus vT) (Fig. 2B, Table S2). Most of the DEGs were detected along the proximal-distal axis of the PE, whereas the differences between dorsal and ventral positions were most notable in the PE samples located near the optic nerve head (Fig. 2B).

To classify the PE and Me DEGs according to their expression patterns along the two axes of the PE and Me, we performed K-means clustering (K=8) based on the average expression of the 5495 unique collective DEGs (Fig. 2C, Table S3). For the genes with similar expression patterns based on the clustering analysis, we performed gene ontology (GO) functional enrichment analyses (Fig. 2C, Table S4). In Fig. 2D-I, line plots represent the expression patterns of selected key genes belonging to the enriched GO categories.

Cluster 1 (C1, 1154 genes; Fig. 2C) consisted of genes expressed in all of the PE samples but with low/undetectable expression in the Me. C1 was enriched for genes encoding lysosome-associated proteins (Fig. 2C,D, Table S4), including multiple subunits of vacuolar ATPase, a transmembrane complex that mediates vacuolar acidification and is essential for lysosome function. Elevation in lysosomal activity is associated with metabolic demand in PE progenitors, and with the extensive phagocytic activity that is a hallmark of mature RPE (Yang and Wang, 2021). Some of these lysosome-associated genes likely reflect the biogenesis of melanosomes, lysosome-related organelles generated in the embryonic PE (Wiriyasermkul et al., 2020). Among the genes required for melanosome maturation are those encoding the membrane transport proteins that control osmolarity, pH neutralization (*Slc24a5*, *Slc45a2*, *Oca2*) and cysteine import (*Mfsd12*), and enzymes involved in pigment biogenesis (*Tyr*, *Tyrp1*, *Dct*; Fig. 2E).

Whereas melanosome biogenesis is already occurring in all of the PE positions at this stage, other properties of mature RPE are acquired gradually, as evidenced by the DEGs in clusters 2 and 3 expressed in a PE-O^high^T^low^ gradient, resembling the expression pattern of *Sox9* (C2 and C3, 1048 genes; Fig. 2C). These clusters were enriched for genes related to the extracellular matrix and basement membrane proteins, many of which are components of Bruch's membrane, a collagen-rich extracellular matrix layer that contributes to the selective barrier function of the RPE and forms from both RPE and Me (Bai et al., 2009; Booij et al., 2010b) (Fig. 2F). The PE-O^high^T^low^ DEGs were further enriched for fatty acid metabolic processes, including cholesterol metabolism, transporters, and enzymes required for the biogenesis of long-chain fatty acids (Fig. 2G). In the adult, the RPE generates long-chain fatty acids and transports them to the photoreceptor's outer segments (Jun et al., 2017; Kautzmann et al., 2020). The expression pattern of these transporters and enzymes in the embryo represents RPE maturation through the acquisition of properties required for supporting outer-segment development and maintenance.

The genes in clusters 4 and 5 (C4 and C5, 1162 genes; Fig. 2C) were expressed in an opposite gradient (PE-O^low^T^high^), resembling the pattern of *Pax6* expression. The GO terms that were enriched in this group were predominantly related to glycolysis and positive cell-cycle regulation (*Mcm3-7*, *Mcm10*, subunits of the DNA replication complex; Fig. 2H), reflecting the immature proliferative state of the PE in the distal optic cup.

DEGs with high expression in the Me were clustered into three groups (2131 genes in clusters C6-C8; Fig. 2C). C6 included genes expressed in all Me positions, whereas clusters C7 and C8 were genes expressed in opposing gradients along the proximal-distal axis of the Me. Notably, the DEGs in C7 (Me-O^high^T^low^) were significantly enriched for GO terms related to the regulation of angiogenesis (Fig. 2I), consistent with a central role in the peripheral expansion of the choroid vasculature (Saint-Geniez and D'Amore, 2004). In addition, enrichment for the Wnt signaling pathway was detected for the genes in C8 (Me-O^low^T^high^). The detection of Wnt-pathway genes in the peripheral Me is in accordance with previous reports on the activity of this pathway in regulating periocular Me differentiation (Carpenter et al., 2015; Kumar and Duester, 2010). This further supports the documented role of the canonical Wnt pathway in RPE differentiation (Fujimura et al., 2009; Westenskow et al., 2009).

The enriched terms for most Me DEGs (C6) were related to extracellular matrix assembly, focal adhesion, and muscle cell differentiation. These results reflect the complexity of the ocular Me, which consists of a heterogeneous population of progenitors related to multiple lineages, including the sclera, choroid vasculature and muscle tissue, together with migrating neural crest cells that give rise to choroidal melanocytes and others that are destined to populate the corneal and iris stroma.

### The TF networks (regulons) in differentiating RPE and ocular mesenchyme

**Fig. 3.**
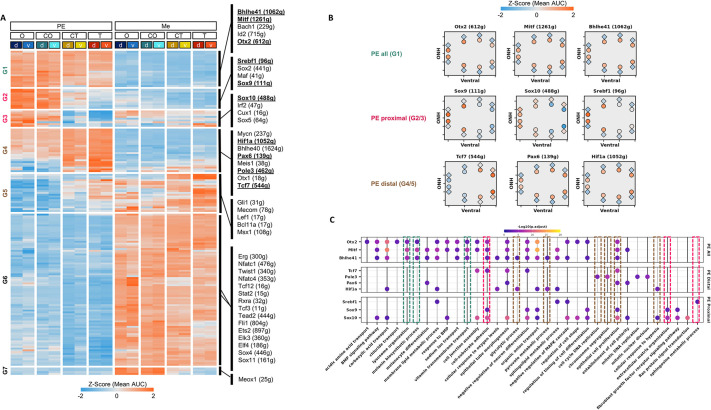
**Gene regulatory networks (regulons) during PE and Me differentiation.** (A) Heat map representing the unsupervised clustering results based on the average activity levels (AUC scores) of each regulon in each sample generated by SCENIC. Selected regulons from each cluster are highlighted. Positions along the proximal (O, position close to optic nerve head) to distal (T, tip of the optic cup, close to lens) axes of the optic cup are indicated. The central positions are termed CO and CT (see Fig. 1I). g, genes; d, dorsal; v, ventral. (B) Football plots representing the average regulon activity (AUC score) pattern of the indicated regulons. g, genes; ONH, optic nerve head. (C) Bubble plot representing the functional enrichment results for the selected indicated regulons.

To identify transcriptional regulators and their potential targets in the developing PE and Me, we performed regulon analysis using SCENIC (single-cell regulatory network inference and clustering; Aibar et al., 2017). This analysis, conducted on the 5495 unique DEGs, identified 173 regulons, each representing a module of a TF and its predicted targets based on co-expression and detection of its binding motif in upstream regulatory regions. We then conducted hierarchical clustering based on the mean spatial regulon activity, generating seven regulon groups (G1-7), each representing a network of regulatory factors with a similar pattern of activity (Fig. 3A, Tables S5, S6).

For the regulons that were active in the PE, we detected three main patterns: highly active regulons in all PE positions with low activity in the Me (PE all, G1), and those with PE-O^high^T^low^ (PE proximal, G2 and G3) or PE-O^low^T^high^ (PE distal, G4 and G5) activity patterns (Fig. 3A). The average activity patterns of selected PE regulons from each group are presented as football plots (Fig. 3B).

Among the regulons in G1 were members of the MiT family of basic helix-loop-helix (bHLH) leucine zipper TFs: MITF (1261 genes) and BHLHE41 (1062 genes) (Fig. 3A,B), which are considered to be functionally redundant and to regulate the melanogenic and phagosomal gene networks (Bharti et al., 2012). Accordingly, 65% of the predicted MITF targets were also detected as BHLHE41 targets (819 genes), including genes involved in lysosome organization, melanin biosynthesis and vitamin transmembrane transport (Fig. 3C). Interestingly, predicted targets for OTX2 were enriched for similar GO terms (Fig. 3C). This overlap in targets supports earlier findings on the physical association between MITF and OTX2 and their co-regulation of RPE genes (Fan et al., 2018; Martinez-Morales et al., 2003).

Additional TFs with similar activity patterns included ID2 (715 genes), a member of the inhibitor of DNA-binding proteins (ID) family, which is important for regulation of cell proliferation and is involved in oxidative injury in RPE cells, and the BTB and CNC homology 1 TF BACH1, which is involved in the response to oxidative damage (Fan et al., 2018; Tan et al., 2013) (Fig. 3A).

The regulons that exhibited O^high^T^low^ activity patterns in the PE included SOX9 (111 genes), a SOXE family member that functions in RPE maturation (Cohen-Tayar et al., 2018; Goto et al., 2018), and SOX10, another member of the SOXE family (448 genes) (Fig. 3A,B). SOX10 functions with SOX9 in oligodendrocyte and melanocyte differentiation (Finzsch et al., 2008; Harris et al., 2013), supporting their predicted shared activity in the PE, with 69% of predicted SOX9 targets overlapping with those of SOX10. Furthermore, among the predicted targets of SOX10 and SOX9 were extracellular matrix and cell-substrate adhesion-encoding genes, suggesting a role for these TFs in regulating RPE morphology and adhesion (Fig. 3C). Another regulon with a similar activity pattern was the sterol regulatory element binding transcription factor 1 (SREBF1, 96 genes; Fig. 3A,B), a key factor in fatty acid and lipid biosynthesis with an as-yet unknown role in RPE development (Brown and Goldstein, 1997). The SREBF1 regulon was enriched for the sphingosine metabolic process, suggesting that membrane lipid metabolism plays a role in RPE differentiation (Fig. 3C).

The group of regulons that exhibited the O^low^T^high^ activity pattern in the PE with variable activity in the Me (Fig. 3A,B, G4) included PAX6 (139 genes) and OTX1 (18 genes), which are known regulators of early stages of RPE differentiation and of development of the pigmented lineages of the iris and ciliary body, respectively (Cohen-Tayar et al., 2018; Martinez-Morales et al., 2001; Raviv et al., 2014; Trimarchi et al., 2009). Similarly, the activity pattern of TCF7 (544 genes), a key mediator of the Wnt signaling pathway, agreed with the role of Wnt signaling in neural and peripheral optic cup lineages (Fig. 2C; Fuhrmann, 2010). Several other factors with as-yet-unknown roles in RPE differentiation also exhibited PE-O^low^T^high^ activity and are therefore predicted to control cellular and biological pathways in PE progenitors; among these were MEIS1 (Dupacova et al., 2021; Hisa et al., 2004), and the hypoxia-inducible transcription factor 1a (HIF1a, 1052 genes), a key regulator of oxygen adaptation, involved in RPE survival and development of the choroid vasculature, retina, and anterior auxiliary structures of the iris and ciliary body (Lange and Bainbridge, 2012; Lange et al., 2012) (Fig. 3A,B). In addition, several glycolytic genes were predicted targets of HIF1a in the developing RPE, implicating a role for this factor in regulating the metabolic state of PE progenitors (Fig. 3C).

The Me regulons were primarily active throughout the Me positions (G6), although some exhibited differential activity in the proximal regions (G7). The main Me regulons included the nuclear factor of activated T-cells (NFAT) family (NFATC1 and NFATC4) and members of the E-twenty-six (ETS) family (ETS1, ETS2, FLI1). Both families have an established role in regulating angiogenesis (Bretz et al., 2013; Wei et al., 2009; Toyama et al., 2017) (Fig. 3A).

Overall, the regulon analysis identified the known TFs in RPE differentiation and indicated the TF family members and previously unidentified factors that regulate the networks involved in different stages of RPE differentiation. The results provide a valuable resource to probe gene expression patterns and regulatory pathways dictating the stepwise differentiation process of the PE and of multiple lineages that are derived from the ocular Me.

### SWI/SNF complexes are required for the expression of the RPE regulons, inhibition of cell proliferation, and repression of neural genes

**Fig. 4.**
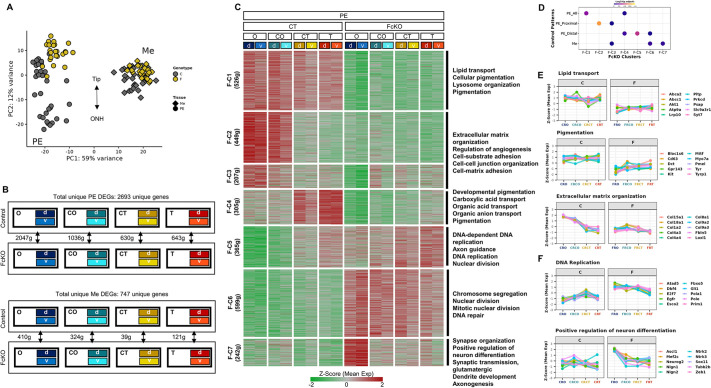
**SWI/SNF complexes control the RPE differentiation program while inhibiting the onset of neural progenitor genes.** (A) PCA plot based on library size normalization from DESeq2 for the control and FcKO samples (yellow, FcKO; black, control; circle, PE; diamond, Me). (B) Scheme describing the statistical comparisons analyzed by DESeq2 between control and FcKO PE samples, with the number of differentially expressed genes (DEGs) for each comparison [|log2(fold change)|>1, *P-*adj<0.05]. (C) Heat map (K-means, K=7) of clustering analysis based on the average positional expression of all DEGs in FcKO versus control. On the right are several selected enriched GO terms for each set of clusters with similar patterns. (D) Bubble plot showing the enrichment of FcKO clusters relative to control clusters. (E,F) Line plots showing the proximal-distal expression pattern of selected genes from the enriched GO terms of downregulated (E) and upregulated (F) clusters. Positions along the proximal (O, position close to optic nerve head) to distal (T, tip of the optic cup, close to lens) axes of the optic cup are indicated. The central positions are termed CO and CT (See Fig. 1I). d, dorsal; v, ventral; g, genes.

We next characterized the transcriptional changes in the PE and Me following SWI/SNF inactivation. PCA of the Geo-seq samples presented a reduction in spatial heterogeneity for the FcKO PE compared with the distribution of the control samples (Figs 1K, 4A). We then performed DEG analyses comparing the transcriptome in the corresponding proximal-distal positions of the FcKO and control PE and Me [|log2(fold change)|>1, *P-*adj<0.05]. For the PE samples, we identified 2047, 1036, 630 and 643 DEGs in the O, CO, CT and T regions, respectively (2693 unique DEGs; Fig. 4B, Table S7). For the Me samples, we identified 747 DEGs, most of them in the O and CO positions (Fig. 4B, Table S7).

These analyses suggested that the SWI/SNF complexes are essential for the PE transcriptome and that this activity is more prominent in PE cells located in the proximal regions of the optic cup, which are in a more advanced stage of RPE differentiation. The adjacent Me lineages, primarily in the proximal optic cup, showed a non-cell-autonomous response to the phenotype of the FcKO PE.

K-means clustering (K=7) of the mean positional expression levels of the DEGs in the PE (Fig. 4C, Table S8) revealed that most of the downregulated genes in the FcKO (F-C1–F-C3, 1182 genes, ∼80%) were significantly enriched in the gene clusters expressed in the controls of either all of the PE (F-C1) or PE proximal (O^high^T^low^, F-C2/F-C3; Fig. 4D) regions. GO functional enrichment analysis (Table S9) showed that these downregulated genes are enriched, as in the respective clusters of the control, for pigmentation, lysosome organization and lipid transport (F-C1), extracellular matrix organization and cell adhesion (F-C2 and F-C3; Fig. 4C). Examples of downregulated genes from the GO terms are shown in line plots (Fig. 4E). The remaining 20% of the downregulated DEGs in the FcKO PE (F-C4, 305 genes) were enriched for genes with higher expression in the distal region of the control (O^low^T^high^, F-C4/F-C5; Fig. 4D). These genes were enriched for terms related to pigmentation and ion transporters (Fig. 4C).

We further detected 1206 genes that were upregulated in the FcKO PE (F-C5–C7). Cluster F-C5 (Fig. 4, 365 genes) represented genes with distal expression patterns in the control (O^low^T^high^) that increased in the proximal position of the FcKO PE. These genes were enriched for terms related to DNA replication and cell proliferation (Fig. 4C,F). The genes in F-C6 (599 genes) had low expression in the control PE and were upregulated in all positions of the FcKO PE. These genes were enriched for terms involved in mitotic nuclear division and DNA repair (Fig. 4C). Finally, expression of the genes in F-C7 (242 genes) was low or not detected in the control PE, but ectopically expressed in the FcKO PE, specifically in the proximal O region. GO enrichment analyses for these genes showed enrichment for terms related to neural differentiation (*Neurog2*, *Sox11*, *Ascl1*, *Pou3f2*), axonogenesis, and synaptic organization [*Tubb2b*, *Nefm*, *Map2*, *Cdh2*; Fig. 4C,F], thus supporting partial transition from PE to the neuronal differentiation program.

**Fig. 5.**
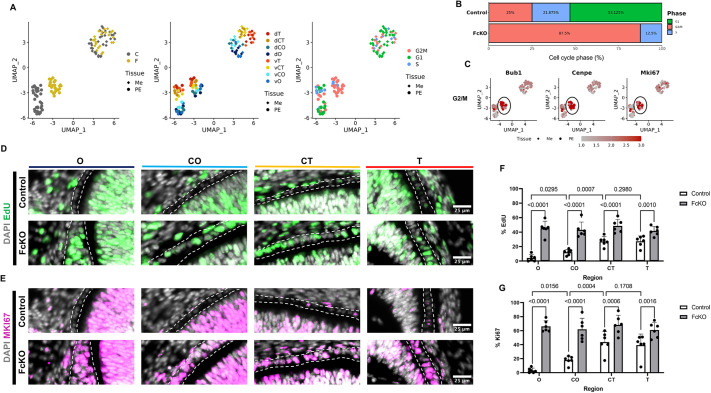
**SWI/SNF complexes inhibit cell proliferation in RPE development.** (A) UMAP plots based on the library size normalization generated by Seurat, colored by genotype (left), position (middle) and cell-cycle state (right). (B) Bar plot describing the assigned cell-cycle phase for each genotype, based on Seurat analysis. (C) UMAP plots colored by the normalized expression levels of *Bub1*, *Cenpe* and *Mki67* (their expression levels in FcKO PE samples is encircled). (D,E) Representative images of EdU^+^ cells (E14.5) (D) and MKI67 (E) detected by antibody labeling in central to peripheral regions of the PE of control (top row) and FcKO (bottom row). Dashed lines delineate their PE. (F,G) Quantification of the percentage of EdU^+^ (F) and Ki67^+^ (F) cells out of total DAPI^+^ cells. The *P*-values were calculated by one-way ANOVA and corrected with Tukey's test for multiple comparisons (*n*=6). Error bars represent s.d. Position along proximal (O, position close to optic nerve head) to distal (T, tip of the optic cup, close to lens) axes of the optic cup are indicated. The central positions are termed CO and CT (see Fig. 1I). d, dorsal; v, ventral.

The above clustering analysis indicated major alterations in cell-cycle regulation following inactivation of the SWI/SNF complexes in the developing RPE. Accordingly, the Seurat cell-cycle scoring analysis (Butler et al., 2018) classified most of the samples in the FcKO as G2/M (87.5%) or S phase (12.5%), whereas the control samples were classified as G1 phase (∼50% of all samples; Fig. 5A,B). The expression patterns of three G2/M genes further illustrate this transition (*Bub1*, *Cenpe* and *Mki67*; Fig. 5C).

To validate further the cell-cycle changes in the PE of the FcKO, we quantified the percentage of cycling cells (by antibody labeling to MKI67) or cells in the S phase [by 5-ethynyl-2′-deoxyuridine (EdU) pulse labeling] from DAPI^+^ cells in regions corresponding to the spatial transcriptome analyses (Fig. 5D-G, Fig. S3A). In the controls, supporting the central-to-peripheral progression of RPE maturation, we detected a significantly higher percentage of proliferating cells (EdU^+^ and MKI67^+^) in the distal PE regions (CT, T) compared with the proximal region (O). These regional differences in cell proliferation were diminished in the FcKO PE, where we detected a significant increase in the percentage of both EdU^+^ and MKI67^+^ cells in all spatial positions along the proximal-distal axis of the optic cup (Fig. 5D-G). The increase in proliferation corresponded with the increasing number of cells in FcKO PE (Fig. S3B). Overall, these results support a key role for SWI/SNF in the inhibition of cell proliferation during RPE differentiation.

**Fig. 6.**
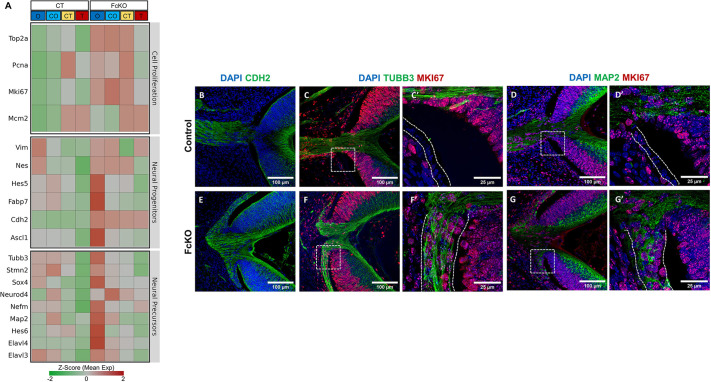
**SWI/SNF complexes inhibit proliferation and neuronal gene expression in the developing RPE.** (A) Heat map presenting the expression levels of genes associated with cell proliferation, neural progenitors and neuronal precursors in the distal to proximal regions of the control and FcKO PE. (B-G′) Antibody labeling of E14.5 control (B-D; C′ and D′ are higher magnification of the respective boxed regions) and FcKO (E-G; F′ and G′ are higher magnification of the respective boxed regions) sections for detection of CDH2 (B,E), TUBB3 and MKI67 (C,F), and MAP2 and MKI67 (D,G). Dashed lines in C′,D′,F′ and G′ delineate the PE.

In addition to the increase in cell-proliferation genes, we detected increased expression of neural progenitor and neural precursor genes in the FcKO PE, including *Cdh2* (N-cadherin), *Map2*, *Nefm* (also known as *Nf165*) and *Tubb3* (Fig. 6A). This suggests transition of the PE to a neural progenitor/precursor-like program. To evaluate this possibility further and to examine the cell-cycle status of aberrant PE cells in the FcKO, we used antibody labeling to examine the expression of CDH2, NEFM and MAP2 in control (Fig. 6B-D′) and FcKO (Fig. 6E-G′) E14.5 eyes. These proteins were below detection levels in the control embryonic PE (Fig. 6B-D′) but were either detected in all FcKO PE positions (CDH2; Fig. 6E) or increased in the proximal positions (TUBB3, MAP2; Fig. 6F,G). Notably, TUBB3 and MAP2 were detected in MKI67^+^ cells within the proximal region (Fig. 6F,F′,G,G′). These findings support a role for the SWI/SNF complexes in inhibiting the neurogenic program in the PE. Moreover, the detection of neuronal markers together with cell-proliferation markers suggests that some of the SWI/SNF-deficient neural precursors failed to exit the cell cycle. Together, these results show that the SWI/SNF complexes inhibit neural progenitor genes in the PE lineage and are required for these cells' exit from the cell cycle.

**Fig. 7.**
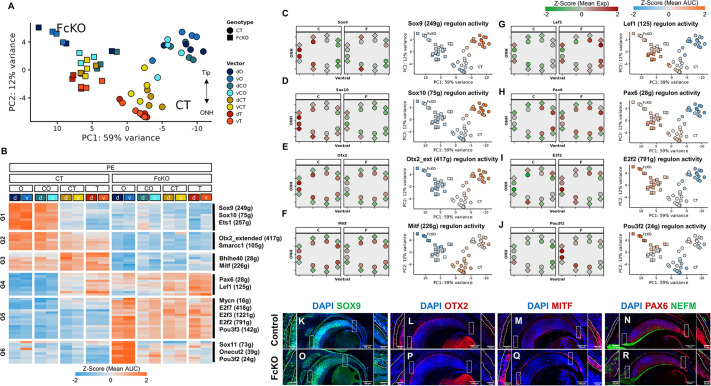
**SWI/SNF complexes control the PE gene-regulatory networks.** (A,B) PCA plot based on the regulon activity scores (A) and heat map of unsupervised clustering analysis with selected regulon TFs (B). d, dorsal; v, ventral. Position along proximal (O, position close to optic nerve head) to distal (T, tip of the optic cup, close to lens) axes of the optic cup are indicated. The central positions are termed CO and CT (see Fig. 1I). d, dorsal; v, ventral. (C-J) Football plots (left) and PCA plots (right) illustrating the relative expression and regulon activity of *Sox9* (C), *Sox10* (D), *Otx2* (E), *Mitf* (F), *Lef1* (G), *Pax6* (H), *E2f2* (I) and *Pou3f2* (J). (K-R) Antibody labeling counterstained with DAPI of E14.5 control (K-N) and FcKO (O-R) sections for SOX9 (K,O), OTX2 (L,P), MITF (M,Q) and PAX6 and NF165 (NEFM) (N,R). ONH, optic nerve head; g, genes.

To identify the key TFs and transcriptional networks that depend on the SWI/SNF complexes in the PE, we implemented SCENIC analyses on the DEGs identified in the FcKO PE (Tables S10, S11). A PCA plot based on the regulon activity scores showed reduced heterogeneity in most of the FcKO PE samples (Fig. 7A). Moreover, unsupervised clustering based on the average positional regulon activity scores highlighted the downregulated (G1-G3) and upregulated (G4-G6) regulons in the FcKO versus control PE (Fig. 7B).

Among the downregulated regulons (G1-G3), transcripts of both *Sox9* and *Sox10* were reduced, suggesting that they are transcriptional targets of SWI/SNF (Fig. 7C,D). Interestingly, *Otx2* and *Mitf* transcripts were not eliminated in the FcKO samples (Fig. 7E,F), inferring an additional level of regulation of their activity by the SWI/SNF complexes.

The regulon clusters in which activity was higher in the FcKO compared with control PE (Fig. 7B, G4-G6) included *Lef1* and *Pax6*, two genes normally expressed in the distal positions, activity of which spread to central positions in the FcKO PE (Fig. 7B,G,H). We also observed increased activity of several TFs involved in cell proliferation, including *E2f2* and *E2f3*, consistent with the observed aberrant cell-cycle regulation in the FcKO PE (Figs 5, 7B,I). Finally, regulons of several neuronal factors – *Sox11*, *Onecut2* and *Pou3f2* – were upregulated specifically in the proximal region of the FcKO PE, supporting the cryptic expression of neuronal genes in SWI/SNF-deficient RPE cells (Fig. 7B,J).

The observed reduction in regulon activity suggested that SWI/SNF is required for the upstream transcription of TFs, or, alternatively, for TF activity on their targets. To discern between these options, we characterized the TFs' distribution by antibody labeling in control and FcKO developing eyes (Fig. 7K-R). This analysis confirmed reduction of the SOX9 protein corresponding with the reduced transcript (Fig. 7C) in the FcKO compared with the control (Fig. 7K,O). Unlike its RNA levels (Fig. 7E), OTX2 protein was also eliminated from the FcKO PE, where its normally expressed (Fig. 7L,P). In contrast, MITF protein was maintained in the FcKO PE (Fig. 7M,Q). These results suggested that the expression of some TFs is dependent on the SWI/SNF complexes at the transcription level (*Sox9*), and that of others depends on these complexes for expression and possibly also protein stability (OTX2), and yet other TFs require SWI/SNF for their activity on tissue-specific genomic targets (MITF).

Antibody labeling further confirmed the ectopic expression of neuronal and cell-cycle factors; PAX6, normally expressed in RPCs and restricted to the distal PE at E14.5, was detected in the proximal positions of the FcKO PE (Fig. 7N,R). The elevation in neural progenitor/precursor genes in the FcKO PE was not accompanied by cryptic expression of genes encoding the retinal TFs *Rax* and *Six6* (Fig. S4A,B). We also did not detect, by antibody labeling, increased expression of the early retinal determinant VSX2, a TF expressed in RPCs and bipolar cells (Levine and Green, 2004; Liu et al., 1994) (Fig. S4C,D), the ganglion cell TF POU4F2 (also known as BRN3b), or the ganglion, amacrine and bipolar cell marker ISL1 (Gan et al., 1996; Martin-Partido and Francisco-Morcillo, 2015) (Fig. S4E-H). Taken together, these findings suggest that the SWI/SNF complexes inhibit the expression of neural progenitor/precursor genes in the developing RPE.

## DISCUSSION

**Fig. 8.**
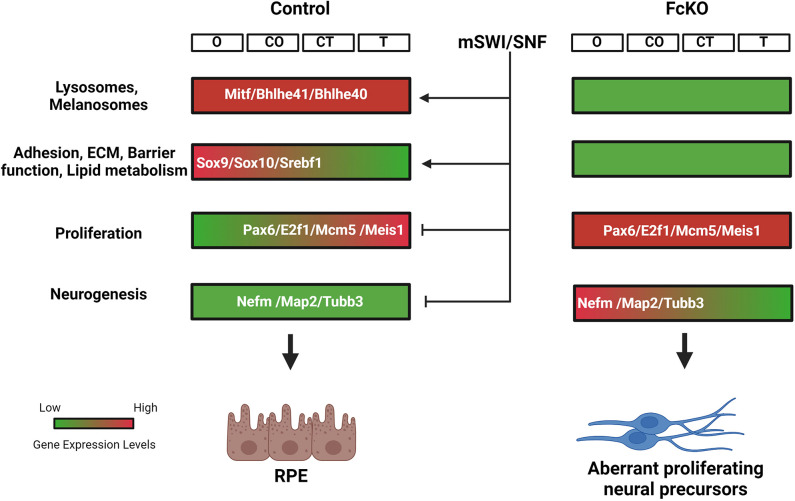
**Schematic of the cellular processes occurring during the gradual differentiation of RPE and the roles of the SWI/SNF complexes.** The key TFs controlling the expression of the RPE differentiation genes are dependent on the SWI/SNF complexes for their expression/activity. At the same time, SWI/SNF complexes inhibit both cell proliferation and the expression of neural progenitor/precursor genes. ECM, extracellular matrix; mSWI/SNF, mouse SWI/SNF. Position along proximal (O, position close to optic nerve head) to distal (T, tip of the optic cup, close to lens) axes of the optic cup are indicated. The central positions are termed CO and CT (see Fig. 1I).

The presented high-resolution spatial transcriptomic atlas of the developing RPE and adjacent ocular Me in control and SWI/SNF-deficient RPE reveals the major TFs and their gene regulatory networks that function during the differentiation of these ocular cell types. The study further illustrates that the RPE-specific gene regulatory networks are dependent on the activity of the SWI/SNF complexes, which are required for the expression and activity of RPE TFs and, at the same time, for the inhibition of the neural progenitor and cell proliferation genes (Fig. 8). The results place SWI/SNF at the intersection between RPE and neural cell fates and the regulation of cell-cycle exit and differentiation.

### Spatial transcriptomic atlas of the gene regulatory networks controlling the gradual differentiation program of the RPE and ocular Me

RPE, an important tissue for retinal function and survival, is also an excellent model to study the regulatory networks that control tissue differentiation. This is because, *in vivo*, the RPE progenitors' spatial position correlates with their stage of differentiation. Geo-seq technology is advantageous for characterizing the developing RPE as it provides a means of obtaining high-resolution transcriptomic data while preserving the positional information, in contrast to single-cell technologies, for which sequencing depth is low and spatial resolution is inferred based on clustering analyses. Indeed, analysis of a single embryonic stage, as shown in this study, exposes the massive changes that occur as this tissue matures, providing information on the expression pattern of any gene of interest during the dynamic stage of PE and Me differentiation. Importantly, the bioinformatics analyses provide further insight into the regulatory networks controlling this gradual differentiation program. The transcriptomic data, which preserve the spatial coordinates, provide a basis for future studies implementing single-cell technologies, which will allow positional information to be attributed to the identified cell populations.

The RPE originates from the bipotential neuroectoderm of the optic vesicle. This bifurcation, recently characterized using single-cell sequencing in zebrafish embryos (Buono et al., 2021), is the first step in cell-fate decisions of the optic neuroectoderm. However, the subsequent differentiation and tissue maturation occur rapidly in the fast-developing teleost, thus precluding insights into the subsequent transcriptional transitions occurring during tissue maturation. The analyses presented in the current study, conducted in the more slowly developing mouse embryo, allowed characterization of the dynamic transcriptional changes during mammalian tissue maturation *in vivo*, revealing the roles of chromatin-remodeling complexes in this developmental transition.

At the stage at which the analyses were performed (E14.5), all of the PE cells have begun to differentiate, based on the detection of multiple genes required for melanosome and pigment biogenesis throughout the optic cup. The transcriptomic data show that some properties and characteristics of the RPE are acquired more gradually in defined spatial patterns. An important RPE activity that is gradually acquired at this stage is the formation of the blood–retina barrier. This is evident from our transcriptomic data; we detected enrichment for genes encoding cell–junction assembly complexes in the proximal (O and CO) regions, including the tight junction-encoding claudin and occludin genes *Cldn1*, *Cldn2*, *Cldn10* and *Ocln* (Feldman et al., 2005; Konari et al., 1995), and junctional adhesion molecule-3 (*Jam3*) (Fig. S5A). Another key structure for the barrier function is Bruch's membrane, which forms from both the RPE and Me (Booij et al., 2010a). This five-layer structure is an important component of the blood–retina barrier and, accordingly, its dysfunction is directly linked to multiple types of inherited retinal dystrophies (Fields et al., 2020). The spatial transcriptome analyses reveal the contribution of PE and Me to the components of Bruch's membrane based on the differential expression between the two tissues of *Col9a3*, *Col4a3*, *Col8a1* and *Col18a1* in the PE and *Col5a1*, *Col6a1/2* and *Col3a1* in the Me. The Bruch's membrane components that emanate from the RPE are detected in the O and CO positions, corresponding to the central-to-peripheral pattern of RPE maturation and choroid vasculature development (Fig. S5A) (Bai et al., 2009; Booij et al., 2010a,b).

Detection of the numerous genes that change expression during tissue differentiation allowed us to predict the upstream regulators. Although the SCENIC pipeline is limited to positive regulatory interactions, it does faithfully predict physiologically relevant regulatory interactions, such as the MITF regulon, which includes known targets in the pigmentation and melanosome pathway (Fig. 3). The regulon analyses indicated redundancy among the key transcriptional regulators, as we detected TFs from the same family controlling a similar subset of targets: 65% of MITF targets were shared with BHLHE41, and many of these targets were involved in pigment biogenesis, whereas 69% of the SOX9 targets were shared with SOX10 (Fig. 3). This overlap further supports the current notion, based on studies in fish embryos, of broad redundancy among the main TFs to ensure robust regulation of tissue-specific gene expression (Buono et al., 2021). Future functional analyses of conditional compound allelic mutants of *Sox9* and *Sox10* are required to determine their combined activity.

The timing of differentiation in adjacent lineages is coordinated by multiple signaling pathways during organogenesis. The canonical Wnt (cWnt) signaling pathway plays a pivotal role in the differentiation of pigmented, vascular and retinal lineages (Fuhrmann, 2008; Fujimura, 2016). During the early stages of optic cup formation, the cWnt pathway is triggered in the dorsal PE by ligands secreted from the surface ectoderm (Carpenter et al., 2015; Hagglund et al., 2013). In the PE, cWnt functions upstream of *Otx2* and *Mitf* to trigger the PE differentiation program and inhibit neural gene expression (Fujimura et al., 2009; Westenskow et al., 2009). Our transcriptomic data document higher expression of the cWnt-pathway genes *Wnt2b*, *Tcf7*, *Lef1* and *Axin2* in the peripheral optic cup (Fig. S5B). The transcriptomic data further suggest opposite activity of noncanonical Wnt (ncWnt) signaling in the proximal PE based on the detection of *Wnt5a*, *Fzd5* and *Sfrp2* (Fig. S5B). The ncWnt pathway plays a key role in cell polarity and adhesion, inhibition of the cWnt pathway, and vascular development (Griffin et al., 2011; Loh et al., 2016; Stefater et al., 2011). Thus, the detection of ncWnt genes in the proximal PE, which is in a more advanced stage of differentiation, suggests the involvement of the ncWnt pathway in the acquisition of RPE morphology and maturation.

### SWI/SNF complexes are required for the RPE differentiation program through regulation of the expression and/or activity of key RPE TFs

Conditional inactivation and global transcriptomic analysis exposed several layers of SWI/SNF regulation of the tissue-specific gene regulatory network. First, we detected downregulation at the transcription and/or protein levels of crucial RPE TFs, including SOX9, SOX10 and OTX2 (Figs 7,8), suggesting regulation at the transcriptional or protein level of these TFs by the SWI/SNF complexes, which could account for the global reduction in their respective regulons in the FcKO PE.

Our analyses detected a global reduction of the MITF regulon, although the *Mitf* transcripts and MITF protein were maintained in the FcKO PE (Fig. 7F). Physical interaction between SWI/SNF complexes and MITF has been reported in melanocytes. This interaction is essential for the activation of MITF target genes (Aras et al., 2019; de la Serna et al., 2006). Thus, in addition to direct regulation of TF expression, the SWI/SNF functions in remodeling the cis-regulatory landscape surrounding the TFs' binding sites to allow activation of transcription. These modes of regulation by SWI/SNF complexes have been previously suggested for proneural TFs in the course of neurogenesis in frog embryos, and possibly for steroid hormone receptors' transcriptional activation properties (Hoffman et al., 2018), and may account for changes in the transcriptional activity of those TFs (such as MITF) for which expression is maintained in the FcKO PE but their regulon is reduced.

### SWI/SNF prevents cryptic expression of neural progenitors and cell-cycle genes

In some vertebrate species, the RPE can regenerate neuroretinal tissue following retinal damage (Barbosa-Sabanero et al., 2012; Coulombre and Coulombre, 1965). Transdifferentiation of the RPE into retinal tissue has been documented in mouse embryos that carry a mutation in RPE TF genes, including *Otx2/1* (Martinez-Morales et al., 2001), *Mitf* (Scholtz and Chan, 1987), β-catenin (Westenskow et al., 2009) and *Yap1* (Kim et al., 2016). In contrast to these loss-of-function models, loss of SWI/SNF activity in the mutant RPE resulted in PE cells reverting to neural progenitor features but without the expression of retina-specific markers. Moreover, these aberrant SWI/SNF-deficient neural progenitors initiated the expression of early neural precursor genes, also detected by antibody labeling, including MAP2, TUBB3 and NEFM, although some of these mutant cells failed to exit the cell cycle based on co-expression with MKI67 (Figs 6,8).

These SWI/SNF complex-deficient PE phenotypes are reminiscent of *Drosophila* neuroblasts and mouse cortical progenitors devoid of key SWI/SNF subunits, resulting in differentiation arrest of the neural progenitors and giving rise to pre-malignant aberrant neural precursors (Eroglu et al., 2014; Nguyen et al., 2018). Nguyen et al. further showed that the increase in cell-cycle genes following SWI/SNF cortical inactivation stems, at least in part, from the derepression of cWnt signaling by the SWI/SNF (Nguyen et al., 2018). Upregulation of Wnt-pathway genes was also reported in RPCs following conditional inactivation of *Smarca4* (Aldiri et al., 2015). A complex interplay between SWI/SNF and cWnt may also contribute to the enhanced proliferation observed in the FcKO PE, as we detected increase expression of several cWnt targets: *Axin2*, *Mycn* and *Wif1* (Fig. S5B). Notably, however, the Wnt receptors were overall reduced, suggesting that, in the PE, the SWI/SNF complexes intersect with the cWnt pathway at the level of transcriptional target regulation, similar to the previously proposed SWI/SNF activity in regulating the Wnt–enhanceosome complex in the fly (Collins and Treisman, 2000; van Tienen et al., 2017).

### A role for SWI/SNF in the transition from neuroectoderm to ocular lineages

The apparent fate transition of PE to neuroectoderm following inactivation of the SWI/SNF complexes suggests their requirement for the transition of neural progenitors to the ocular pigmented and retinal lineages. The FcKO PE cells express high levels of *Pax6*, *Lhx2*, and the proneural genes *Pou3f2* and *Ngn2*, yet these aberrant neural cells fail to express early and late retinal progenitor genes (*Vsx2* and *Sox9*) and TFs that are expressed in specified retinal precursors – *Otx2* (photoreceptors, bipolar cells), *Pou4f2* (ganglion cells) and *Isl1* (ganglion, amacrine and bipolar cells). The limited differentiation potential of the FcKO cells infers that the SWI/SNF complexes are needed for expression of both RPE- and retinal-specific genes. In this regard, the deletion of *Smarca4* in RPCs has been documented to result in altered cell-cycle exit and reduced survival of the RPCs, although retinal cell-fate specification was not compromised (Aldiri et al., 2015). In this conditional mutation of *Smarca4* in the retina, partial compensation by *Smarca2* could mask the SWI/SNF requirement for cell-fate specification, which, based on our findings, may also be compromised for the retinal lineages. Thus, SWI/SNF complexes provide an important epigenetic layer of regulation required for the transition from neuroectoderm to ocular lineages of the RPE and most likely also of the retina.

## MATERIALS AND METHODS

### Animals

Mice were kept at the Tel Aviv University Animal House Facility. Animal use was approved by the Tel Aviv University Animal Care and Use Committee (Approval Number 28-02-2021). The analysis was conducted on *Baf155*^*loxP/loxP*^*; Baf170*^*loxP/loxP*^*;Dct-Cre* and control littermates that do not carry the *DCT-Cre* (the genotypes of the GEO-seq samples are listed in Table S1). The *Baf155^loxP/+^, Baf170^loxP/+^* and *Dct-Cre* mouse lines (Choi et al., 2012; Davis et al., 2009; Tuoc et al., 2013a) were maintained on a C57Bl6/J genetic background. The primers used for genotyping are listed in Table S12.

### Geo-seq of embryonic RPE and Me

The spatial transcriptome of the developing ocular PE and the adjacent lineages of the ocular Me was obtained according to the Geo-seq protocol with minor modifications (Chen et al., 2017). Briefly, E14.5 mouse embryonic heads were embedded in OCT (Leica Microsystems, 020108926) and cryosectioned at a thickness of 20 μm. Sections were transferred onto LCM PEN membrane slides, immediately fixed in ethanol, and stained with 1% (v/v) Cresyl Violet acetate in 75% ethanol solution (Sigma-Aldrich). We analyzed one central section of each eye containing the lens and optic nerve head. From the selected sections, using LCM, we isolated eight adjacent samples along the proximal-distal axis from both dorsal and ventral positions of the PE and the adjacent Me (Fig. 1I). The samples, with cutting lengths ranging from 180 to 450 µm, were collected (Table S1) by laser microdissection, and total RNA pellets were dissolved in a lysis solution, followed by reverse transcription using SuperScript II reverse transcriptase (Invitrogen), and whole transcription amplification with KAPA HiFi HotStart ReadyMix (2×; KAPA Biosystems).

PCR products from the LCM samples were used for automated single-cell RNA-seq library construction based on the Bravo robot station (Cui et al., 2019 preprint). Briefly, PCR products were purified using 0.75× AMPure XP beads (Agencourt), quantified with Qubit dsDNA HS Assay Kit (Thermo Fisher Scientific) on an EnVision plate reader (PerkinElmer), and a cDNA library was constructed using the DNA Library Prep Kit V2 for Illumina (Vazyme) and sequenced on an Illumina Nova 6000 instrument using the 150-bp paired-end reads setting.

### Pre-processing of Geo-seq data

Sequencing quality of the raw sequencing data was evaluated by fastp (Chen et al., 2018). Reads were mapped to the mm10 genome assemblies by HISAT2 (Pertea et al., 2016) using default settings. The mapping ratio was calculated based on the number of mapped reads and total reads for each sample.

All mapped reads were processed by StringTie (Pertea et al., 2016) to quantify gene-expression levels [measured as fragment per kilobase per million mapped reads (FPKM)] using default parameters.

### Identification of DEGs and clustering analysis

DEG analysis was conducted in R using DESeq2 (Love et al., 2014) based on the raw count matrix. The count matrix was prefiltered to include only genes with more than 200 counts in at least three samples and then subjected to library size normalization and variance stabilizing transformation (VST). The normalized transformed data were then subjected to dimensionality reduction using PCA, and to correlation analysis following hierarchical clustering using the stats and pheatmap packages in R to assess sample quality and variance. The ‘combat-seq’ function in the sva package (Leek et al., 2012) was used to correct for batch effect derived from embryo variance (control and FcKO samples separately).

To infer all spatial and tissue-level DEGs, the following statistical tests were performed: (1) within each tissue, the most proximal position (O) was compared with all other positions along the proximal-distal axis (CO, CT, T); (2) for each of these positions, a ventral versus dorsal comparison was performed (vO versus dO, vCO versus dCO, etc.); (3) for tissue comparison, we performed a statistical test comparing all PE samples with all ocular Me samples without considering the spatial positions.

Genes with adjusted *P*-value lower than 0.05 and log2(fold change) higher/lower than 0.5/−0.5 were considered DEGs. We then performed a K-means clustering analysis based on the DEGs using the stat package (source) plotted in a heat map presentation using the pheatmap package (source).

To discover the DEGs caused by the *Smarcc1* and *Smarcc2* conditional mutation (FcKO), the FcKO and control samples were compared for every position along the proximal-distal axis, separately for Me and PE. Genes with adjusted *P*-value lower than 0.05 and log2(fold change) higher/lower than 1/−1 were considered DEGs.

### GSEA of tissue comparison

The genes were ordered based on their log2(fold change) from the analysis comparing all Me with all PE samples and were subjected to GSEA using the R package ClusterProfiler (Wu et al., 2021; Yu et al., 2012). A minimum gene set size of 15 and maximum gene set size of 5000 genes were used, with a *P*-value cutoff of 0.05 and default false discovery rate (FDR). The GSEA results were plotted using the R package enrichplot.

### Functional enrichment

All functional enrichment analyses were conducted with the R package ClusterProfiler (Wu et al., 2021; Yu et al., 2012) using the biological process GO data. For *P*-value adjustment, the Benjamini–Hochberg method was used with a *q*-value cutoff of 0.05 and a *P*-value cutoff of 0.01.

### Inference of regulons and their activity

The analysis by SCENIC (Aibar et al., 2017) included: (1) co-expression analysis between TFs and possible targets using the R package GENIE3; (2) cis-regulatory motif enrichment analysis using the R package RcisTarget, revealing a total of 260 regulons; (3) activity score analysis of each regulon in each sample (AUC score) calculated using the R package AUCell. We used the nonduplicated regulon activity matrix (181 regulons) for PCA, following hierarchical clustering based on the average regulon activity of each position using the R package pheatmap.

### Cell-cycle scoring

Normalization, standardization and dimensionality reduction were conducted based on the R package Seurat, standardized as described (https://satijalab.org/seurat/articles/pbmc3k_tutorial.html). To score samples based on their cell-cycle state, we used the human S and G2/M scores provided by the Seurat package, converted them into mouse symbols, and followed the standardized pipeline (https://satijalab.org/seurat/articles/cell_cycle_vignette.html).

### SPOT web application

The SPOT web app was constructed to visualize the spatial transcriptomic data of E14.5 PE and ocular Me from both controls and FcKO mutants. The app was built using the shiny package in R and implemented on our laboratory website (https://shaiovadia.shinyapps.io/NewShiny_Publication/). The SPOT app allows for (1) visualization of the spatial expression of any gene of interest in football plots; (2) gene list clustering and visualization, which allows the user to enter a list of genes and obtain their spatial expression pattern through K-means clustering visualized using football plots.

### Histology, indirect immunofluorescence and EdU analyses

Paraffin sections (10 ​μm) of embryonic heads were used for Hematoxylin and Eosin staining, and indirect immunofluorescence analyses as described previously (Cohen-Gulkar et al., 2023). Briefly, for immunostaining, the sections were treated with Unmasking Solution (Vector Laboratories, H-3300) for antigen retrieval. To decrease nonspecific binding, sections were incubated for 2 ​h in PBSTG (0.2% w/v gelatin, 0.2% v/v Tween 20 in PBS). Sections were then washed in PBS, incubated overnight with a primary antibody at 4°C, washed with PBSTG, incubated with a secondary fluorescent antibody for 2 h, washed three times in PBS and then with PBSTG, and sealed with fluorescent mounting medium containing DAPI (GBI Labs, E19-18). Antibodies are listed in Table S13. Slides were viewed with an Olympus BX61 fluorescence microscope.

EdU (50 mg/kg body weight, LUM-10540) was administered 3 h before sacrifice of the pregnant females. The embryonic heads were fixed with 4% (v/v) paraformaldehyde for 3 h and embedded in paraffin. Sections were incubated for 30 min in a solution containing copper sulfate, Alexa Fluor^®^ 488 (C10337) or sulfo-cyanine 3 azide (A2330), and L-ascorbic acid (A4544) and imaged using an Olympus BX61 fluorescence microscope.

To quantify the percentage of EdU^+^ and MKI67^+^ cells in the central-peripheral axis, the length of the dorsal and ventral RPE was divided into four equal regions along the proximal-distal axis (O, CO, CT, T). For each region, we counted the total number of cells (DAPI^+^) and the number of EdU^+^ or MKI67^+^ cells. For the ventral and dorsal position of each of these regions, we calculated the average percentage of EdU^+^ or MKI67^+^ cells out of DAPI^+^ cells. The statistical analyses were performed using GraphPad Prism 8.0 as detailed in the figure legends.

## Supplementary Material

10.1242/develop.201488_sup1 Supplementary information
