# Supplementary material for: SWI/SNF complexes are required for retinal pigmented epithelium differentiation and for the inhibition of cell proliferation and neural differentiation programs
Source: Development. 2023 Aug 21;150(16):dev201488. doi: 10.1242/dev.201488 (PMC10482007; doi:10.1242/dev.201488)
Supplement: Supplementary information [file develop-150-201488-s1.pdf]

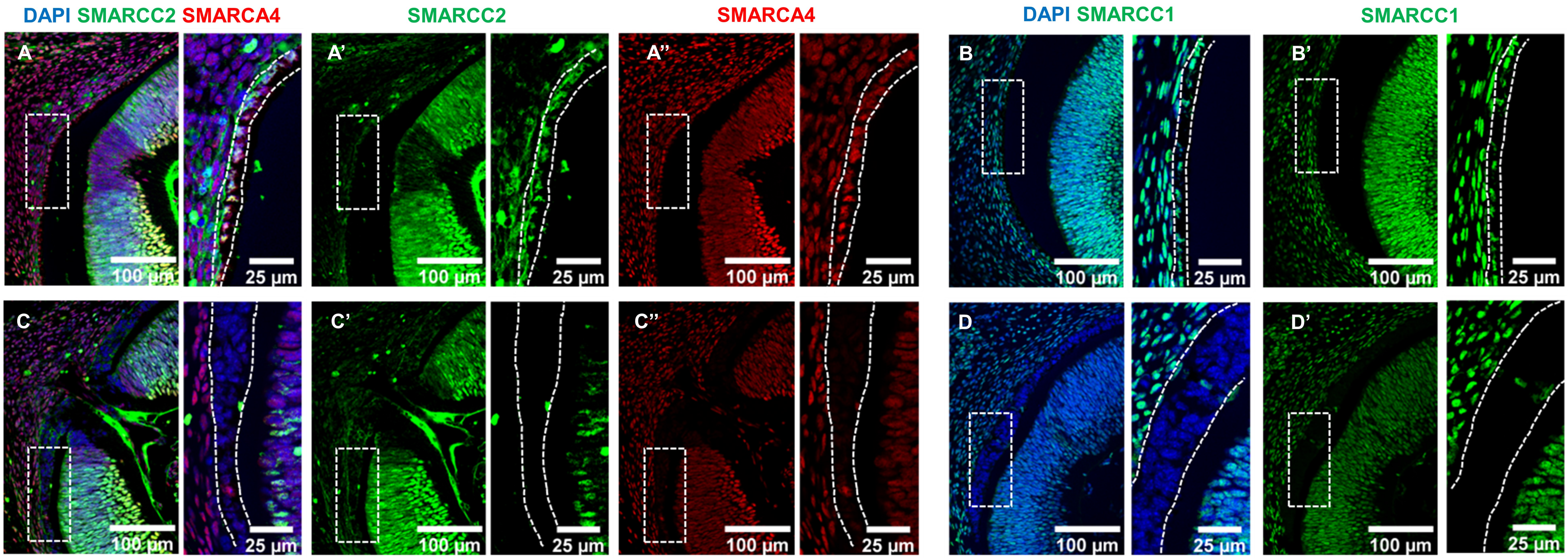

Figure S1

Fig. S1. Higher magnification of the expression patterns of SWI/SNF subunits in the embryonic mouse RPE. Antibody labeling of E14.5 sections (lower magnifications of the labeling are presented in Fig. 1) from control **(A, B)** and FcKO **(C, D)** of SMARCC2 and SMARCA4 (**A, C;** the separate channels shown in **A', A'', C', C''** as indicated) and SMARCC1 (**B, D**; separate channel in **B', D'**). Insets on the right are higher magnifications of the boxed regions. Counterstaining of nuclei with DAPI.

Development • Supplementary information

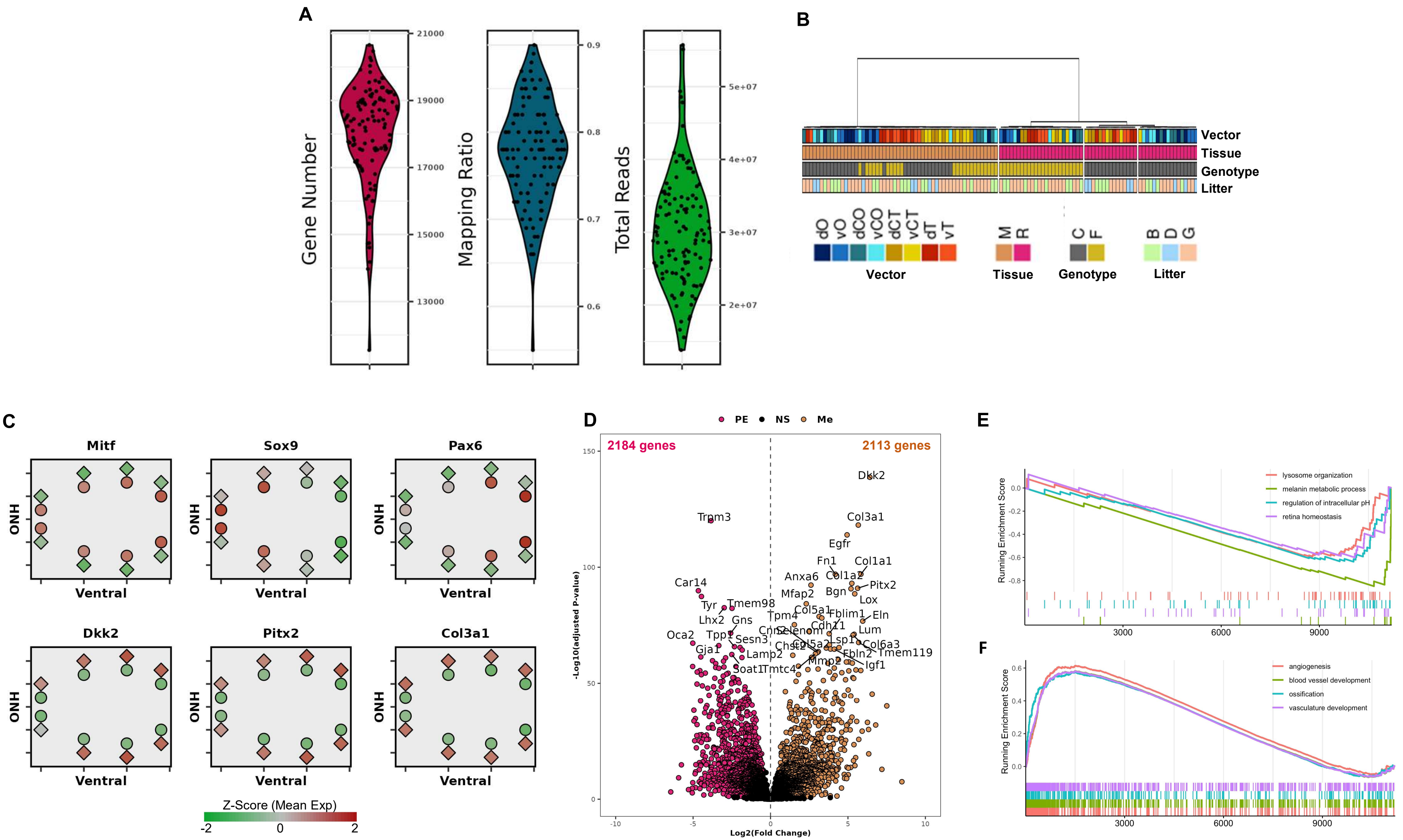
A
Gene Number
Mapping Ratio
Total Reads
B
Vector
Tissue
Genotype
Litter
C
Mitf
Sox9
Pax6
Dkk2
Pitx2
Col3a1
ONH
Ventral
Z-Score (Mean Exp)
D
PE NS Me
2184 genes
2113 genes
-Log10(adjusted P-value)
Log2(Fold Change)
E
lysosome organization
melanin metabolic process
regulation of intracellular pH
retina homeostasis
Running Enrichment Score
F
angiogenesis
blood vessel development
ossification
vasculature development

Fig. S2. Geo-seq data quality control and analyses of E14.5 PE and Me control and FcKO samples. (**A**) Violin plots showing the detected gene number (red), mapping ratio (blue), and total sequenced reads (green) for all samples. (**B**) Annotation tree based on pairwise correlation analysis between all samples. (**C**) Football plot presenting the average relative positional expression of PE (*Mitf, Sox9, Pax6*) and Me (*Dkk2, Pitx2, Col3a1*) genes. Expression levels are represented on a scale of green (low) to red (high). (**D**) Volcano plot representing the differentially expressed genes in control PE vs. Me samples. GSEA plots of overrepresented terms in control PE (**E**) or Me (**F**) genes.

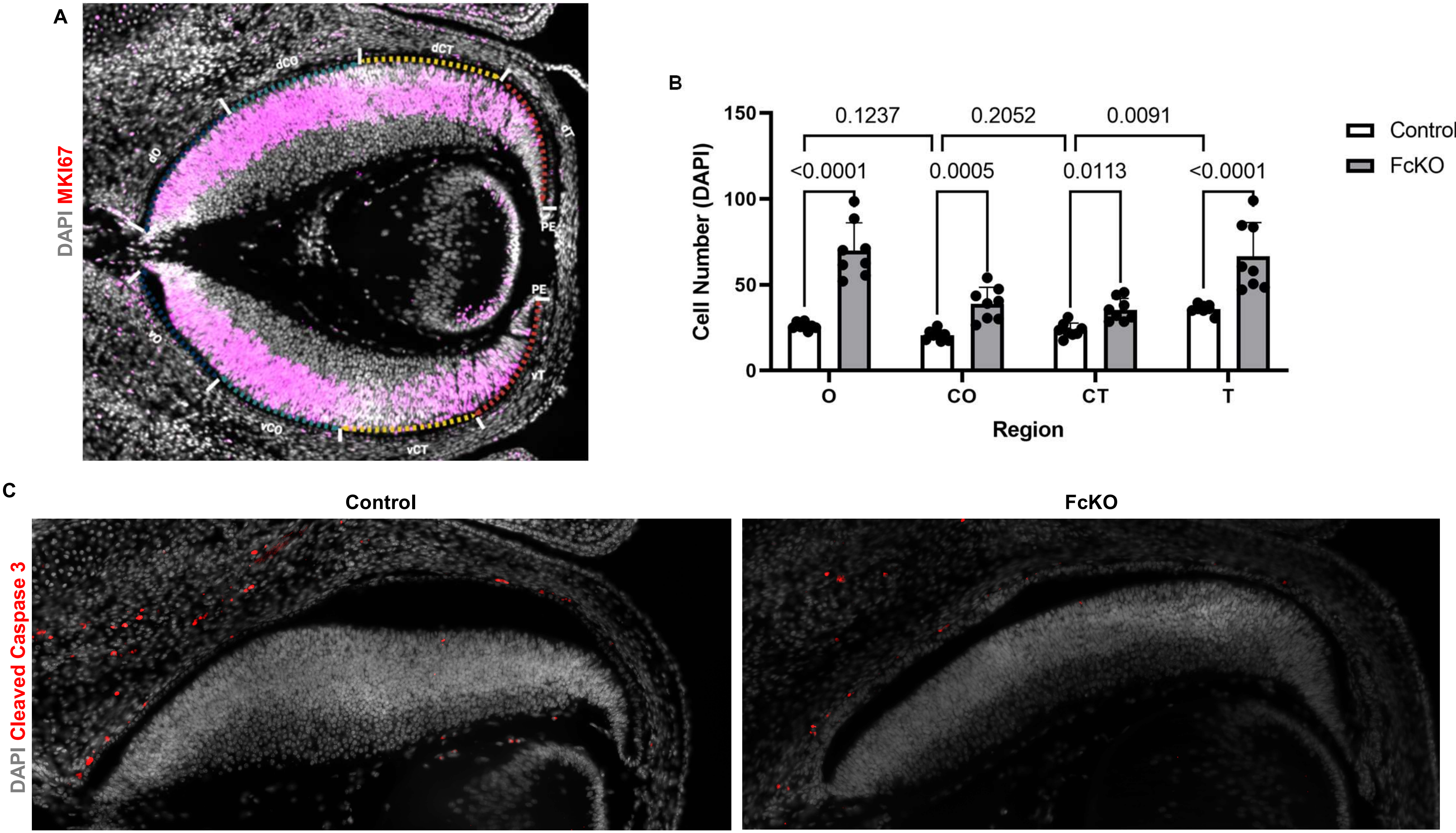

Fig. S3. Quantification of the regional differences in cell numbers, cycle, and survival in FcKO vs. control PE. (A) Example of the PE positions used for quantification of the percentage of MKI67+ and EdU+ cells from DAPI+ cells following antibody labeling on E14.5 eyes. The image is a representative example of a control section labeled with antibodies to MKI67 (counterstaining with DAPI in gray). (**B**) Quantification of the mean cell numbers (DAPI+) in the indicated regions of the control and FcKO optic cups (N = 8). The *p*-values were calculated by one-way ANOVA, and corrected with Tukey's test for multiple comparisons (N = 8). (**C**) Example of Cleaved Caspase 3 detection by antibody labeling of E14.5 control and FcKO samples.

Development • Supplementary information

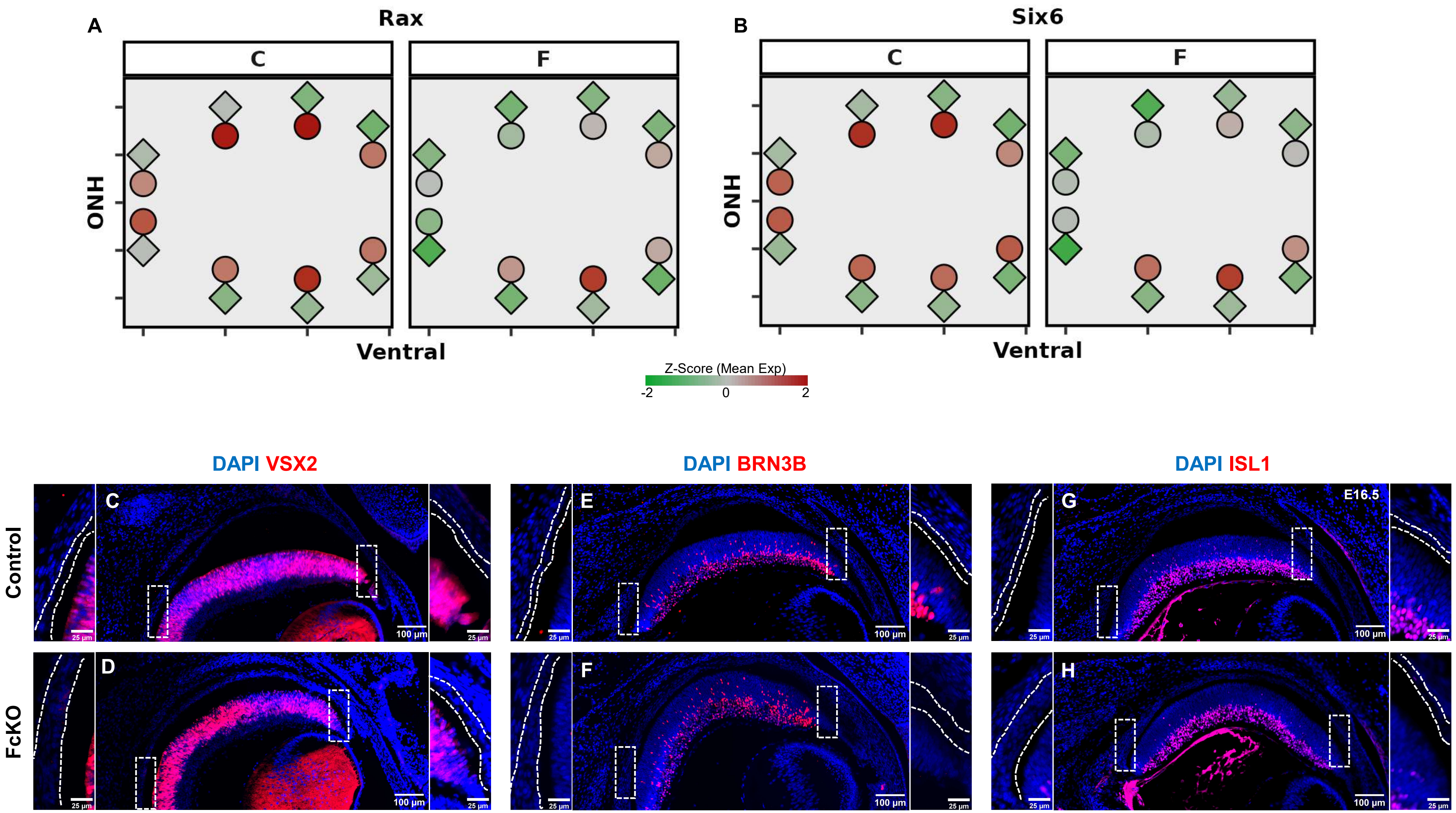

Fig. S4. The expression of several retinal genes is not elevated in the SWI/SNF-deficient PE. Football plots representing the mean relative expression of *Rax* (**A**) and *Six6* (**B**) in the control and FcKO embryos. **(C–H)** Antibody labeling of E14.5 control and FcKO samples, staining for **(C, D)** VSX2**, (E, F)** BRN3b, and **(G, H)** ISL1. Higher magnifications of right and left boxed regions (**C–H**) are presented to the right and left of the main panels, respectively.

Development • Supplementary information

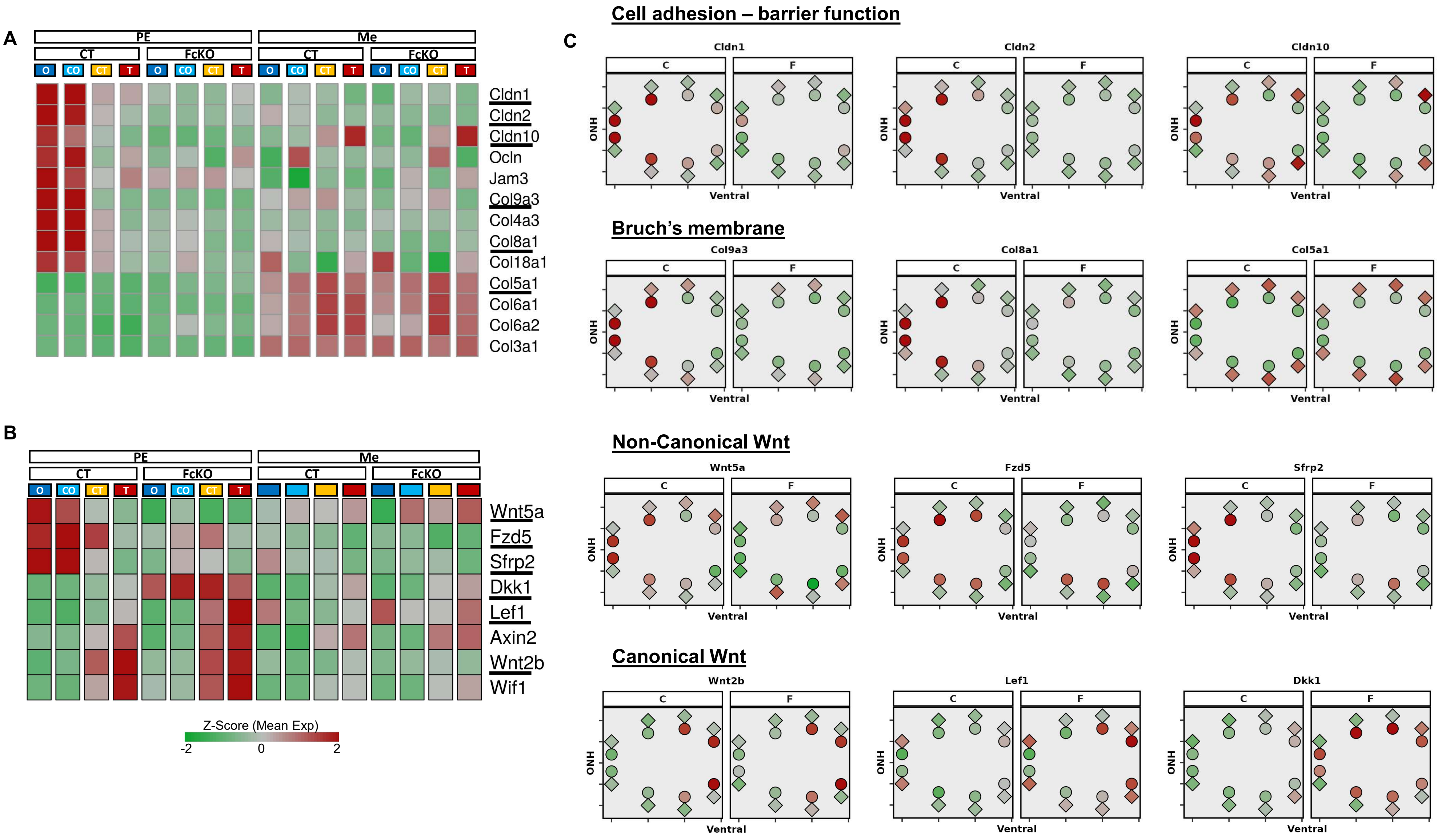
A
PE
Me
CT
FcKO
O
CO
CT
T
Cldn1
Cldn2
Cldn10
Ocln
Jam3
Col9a3
Col4a3
Col8a1
Col18a1
Col5a1
Col6a1
Col6a2
Col3a1
B
Wnt5a
Fzd5
Sfrp2
Dkk1
Lef1
Axin2
Wnt2b
Wif1
Z-Score (Mean Exp)
-2
0
2
C
Cell adhesion – barrier function
Bruch's membrane
Non-Canonical Wnt
Canonical Wnt
C
F
ONH
Ventral

Fig. S5. The change in the expression of cell-adhesion, extracellular matrix and WNT-pathway genes in the control compared to FcKO PE and Me. (**A**) Heat map showing the average spatial expression pattern of genes related to the blood–retinal barrier and Bruch's membrane. (**B**) Heat map showing the average spatial expression pattern of genes associated with canonical and noncanonical WNT signaling. (**C**) Football plots for expression of selected genes from each group in the PE of control and FcKO. ONH, optic nerve head.

Development • Supplementary information

Table S1. Summary of the samples analyzed by Geo-seq. Each tab includes embryo number and genotype and the information on the length of the samples collected by laser-capture microdissection for the Geo-seq analysis.

Click here to download Table S1

Table S2. List of the differentially expressed genes (DEGs) and statistical results based on DESeq2 analysis of the control samples. The DEGs for each indicated comparison are presented in a separate tab.

Click here to download Table S2

Table S3. List of genes in each cluster based on K-means clustering of the control samples.

Click here to download Table S3

Table S4. Functional enrichment results for the genes in each of the presented clusters (*ClusterProfiler*).

Click here to download Table S4

Table S5. List of regulons identified in the control samples based on the SCENIC analysis.

Click here to download Table S5

Table S6. Predicted targets for the regulons identified in control samples by SCENIC analysis.

Click here to download Table S6

Development • Supplementary information

Table S7. List of the differentially expressed genes (DEGs) and statistical results based on DESeq2 analysis of the FcKO compared to control samples. The DEGs for each indicated comparison are presented in a separate tab.

Click here to download Table S7

Table S8. Lists of genes in each cluster based on K-means clustering of the FcKO and control samples.

Click here to download Table S8

Table S9. Functional enrichment results for the genes in each of the FcKO and control clusters (*ClusterProfiler*).

Click here to download Table S9

Table S10. List of regulons identified in FcKO compared to control samples by SCENIC analysis.

Click here to download Table S10

Table S11. Predicted targets for each of the regulons identified by SCENIC analysis in FcKO compared to control samples.

Click here to download Table S11

Table S12. Primers used in this study for genotyping.

Primer	Sequance
Cre F primer	CCG CAG AAC CTG AAG ATG TTC
Cre R primer	TCA TCA GCT ACA CCA GAG ACG
Baf155 F Primer	TGTCATCCATGAGGAGTGGTC
Baf155 R Primer	GGTAGCTCACAAATGCCTGT
Baf170 F Primer	CATGGTGGCTCTCCTAAGCAATCCAA
Baf170 R Primer	CTGGCTTTGTGTGTGTGTGTTTGTTC
Baf170 Mut Primer	GATGCCTGCTTGCCGAATATCATG

Table S13. List of antibodies used in the study.

Click here to download Table S13
