# Supplementary material for: SWI/SNF complexes are required for retinal pigmented epithelium differentiation and for the inhibition of cell proliferation and neural differentiation programs
Source: Development. 2023 Aug 21;150(16):dev201488. doi: 10.1242/dev.201488 (PMC10482007; doi:10.1242/dev.201488)
Supplement: Supplementary file 1 [file develop-150-201488_review_history.pdf]

The SWI/SNF complexes are required for retinal pigmented epithelium differentiation and the inhibition of cell proliferation and neural differentiation programs

Shai Ovadia, Guizhong Cui, Ran Elkon, Mazal Gulkar-Cohen, Nitay Zuk-Bar, Tran Touc, Naihe Jing and Ruth Ashery Padan
DOI: 10.1242/dev.201488

Editor: Francois Guillemot

Review timeline

Original submission:	27 November 2022
Editorial decision:	23 January 2023
First revision received:	21 June 2023
Accepted:	14 July 2023

Original submission

First decision letter

MS ID#: DEVELOP/2022/201488

MS TITLE: The SWI/SNF complexes are required for RPE differentiation and inhibition of neural and proliferation programs.

AUTHORS: Shai Ovadia, Guizhong Cui, Ran Elkon, Mazal Gulkar-Cohen, Tran Touc, Naihe Jing, and Ruth Ashery Padan

I have now received the reports of three referees on your manuscript and I have reached a decision. The referees' comments are appended below, or you can access them online: please go to BenchPress and click on the 'Manuscripts with Decisions' queue in the Author Area.

As you will see, all the referees are enthusiastic about your work, but they also have significant criticisms and recommend a substantial revision of your manuscript before we can consider publication. If you are able to revise the manuscript along the lines suggested, which may involve further experiments, I will be happy receive a revised version of the manuscript. Your revised paper will be re-reviewed by one or more of the original referees, and acceptance of your manuscript will depend on your addressing satisfactorily the reviewers' major concerns. Please also note that Development will normally permit only one round of major revision. If it would be helpful, you are welcome to contact us to discuss your revision in greater detail. Please send us a point-by-point response indicating your plans for addressing the refereeâ€™s comments, and we will look over this and provide further guidance.

Please attend to all of the reviewers' comments and ensure that you clearly highlight all changes made in the revised manuscript. Please avoid using 'Tracked changes' in Word files as these are lost in PDF conversion. I should be grateful if you would also provide a point-by-point response detailing how you have dealt with the points raised by the reviewers in the 'Response to Reviewers' box. If you do not agree with any of their criticisms or suggestions please explain clearly why this is so.

© 2023. Published by The Company of Biologists under the terms of the Creative Commons Attribution License (https://creativecommons.org/licenses/by/4.0/).

Reviewer 1

Advance summary and potential significance to field

Using the DCT (dopachrome tautomerase)-Cre driver mouse, Ovadia and colleagues have generated an RPE-specific conditional knockout (cKO) of two members of the SWI/SNF complexes Smarcc1 (encoding Baf155) and Smarcc2 (Baf170) in the mouse and assess retinal pigment epithelial differentiation and proliferation. Furthermore, they apply geographic positional sequencing (Geo-seq) analysis at one embryonic stage (E14.5) when the pigmented epithelium is a single layer surrounding the developing retina, to demonstrate that these chromatin remodelling complexes are necessary for RPE differentiation; with dual loss of gene function, there is increased proliferation and neural differentiation.

Comments for the author

Ovadia and colleagues have generated an RPE conditional KO of two members of the chromatin remodelling complex Smarcc1 and Smarcc2 using the DCT-Cre driver and furthermore use Geoseq, a type of spatial transcriptomics profiling, to assess the RPE regulons and further characterize RPE and neural differentiation programmes and cell proliferation.

Since the manuscript is both unpaginated and the pages lack line numbers, this review will carefully describe where in the manuscript specific and minor concerns should be addressed.

A. Major concerns:
1. Abstract, Introduction and Results (including Figure 1)
It is too preliminary to comment on the tumour suppressive activity of these members of the SWI/SNF complexes based upon current assessment of proliferation and cell cycle using Ki67 immunostaining and application of the R package Seurat, respectively, as well as the thickening of the PE layer as demonstrated in Figure 1A. Similarly, on page 3, paragraph 1, line 6, the reference to tumor-suppressive role should be downplayed based upon the evidence presented. For Figure 1A, the differences in the thicknesses of the peripheral and central PE and NBL (neuroblastic layers) should be quantified.

2. Cell cycle analysis (including Figure 6)
Additional assessment of proliferation and cell cycle progression could be accomplished through use of markers of S-phase (BrdU incorporation 30-45 minutes prior to embryo extraction) and M-phase (Phosphohistone H3 by immunofluorescence or immunohistochemistry). Alternatively, quantification of cell-cycle phases could be assessed by use of flow cytometry.
Furthermore, it is unclear why apoptosis wasn't assessed either by use of expression of activated Caspase-3 in tissue sections or calculation of the sub-G1 peak using flow cytometry. How well does Seurat correlate with methods such as flow cytometry? For Figure 6, are any of the observed differences between controls and full cKO statistically significant?

B. Specific concerns:
1. Methods The authors should provide a rationale for use of the DCT-driver and also spell out dopachrome tautomerase for its first use.

2. Results a. Please explain the opposite expression gradients of Pax6 and Sox9 for the reader.
b. How were specific regulons selected for more detailed presentation of the data and further discussion? Could others been instructive as well, such as Pole3 or Fli1?
c. Isl1 is not retinal ganglion cell specific (page 12, paragraph 1, line 6) although this is correctly explained in the discussion.

3. Discussion a. Please provide a reference delineating the various collagen genes expressed in Bruch's membrane.

C. Minor Concerns
a. Page 2, paragraph 1, line 3: Add "an" prior to "important". Line 13: Add "stage" after "vesicle".
b. Paragraph 2, line 13: Delete "the" prior to "RPE".

© 2023. Published by The Company of Biologists under the terms of the Creative Commons Attribution License (https://creativecommons.org/licenses/by/4.0/).

c. Page 5, paragraph 1 line 8: add ",respectively" after "Me".
d. Page 7, paragraph 2, line 1: Change "transcription" to "transcriptional".
e. Page 10, paragraph 3, line 5: Replace "indicating" with "supporting".
f. Page 15, paragraph 3, line 7: Delete "the" prior to "SWI/SNF".
g. Page 16, paragraph 2, line 7: Add "(ganglion)" after Brn3b since this marker is specific for RGC.
h. Page 17, paragraph 3, line 3: What is meant by "good morphology sections"?

Reviewer 2

Advance summary and potential significance to field

In this manuscript, Ovadia et al used Geo-Seq to analyze the transcriptional profile of the mouse retinal pigment epithelium at E14.5, a stage in which the tissue is undergoing differentiation with a proximal to distal gradient. They further compare the obtained gene expression atlas with an equivalent analysis obtained from embryos in which the components of the SWI/SNF complex have been inactivated in the differentiating RPE. This comparison shows that this chromatin remodeler is implicated in the control of the expression or activity of different transcription factors essential for the acquisition of RPE identity and to control the neural progenitor state, suggesting that this complex operates at the intersection between RPE and neural cell fate in the retina.

Comments for the author

This is an interesting and well conducted study that might be of broad interest to different readers of this journal. On the one side, the study provides an interesting atlas of genes implicated in the proximal to distal differentiation of the RPE, and, on the other, it illustrates the contribution of the SWI/SNF complex to the gene regulatory network implicated in this differentiation. Overall the manuscript is easy to follow and the figures in general support well the described data. The authors however should consider the following points to strengthen some of their statements along the manuscript.

Main issues
It is somewhat surprising that Wnt signalling components are found enriched in C8 a Me cluster. This contrast with the notion that Wnt signalling is implicated in RPE specification (e.g, PMID: 19553286; 19596317). In the discussion in contrast there is mention of Wnt signalling activation in the more proximal RPE regions. The data related to Wnt signalling should be better explained in the results to avoid confusions and considering the early role of this pathway in the activation of several RPE specific TFs.

The authors indicate that cells of the FcKO RPE “transdifferentiated” or de-differentiate. These terms do not seem appropriate: the PE does not acquire RPE characteristic and therefore cannot transdifferentiate or de-differentiate. Quite likely, it simply retains neural progenitor characteristics failing to activate those required for RPE.

The data related to the identity of the FcKO RPE is somewhat confusing. The F-C7 cluster contains genes belonging to synaptic transmission and synaptic organisation, thus suggesting a rather differentiated state. However, the authors suggest the coexistence of a proliferative marker (Ki67) with an axonal protein (TuJ1) and the absence of retinal identity. Can the authors confirm and better document that Ki67+ cells are also TuJ1+? Tuj1 is considered a pos-tmitotic marker and it is possible that the FcKO RPE contains cells at different stages of specification/differentiation. The lack of Vsx2 expression does not completely rule out the lack of retinal identity. Vsx2 and Mitf have been shown to regulate each other expression and the lack of Vsx2 might be somewhat related to an altered cross talk. Are other retinal enriched genes expressed in the FcKO RPE (e.g. Six6, Rx etc)?

The authors have compared dorsal vs ventral RPE cells, reaching the conclusion that there are little differences or at least less than those observed along the proximo-distal axis. This is somewhat in contrast with the notion that the eye acquires a clear dorso-ventral polarity. It might be interesting to at least discuss this point.

© 2023. Published by The Company of Biologists under the terms of the Creative Commons Attribution License (https://creativecommons.org/licenses/by/4.0/).

The authors justified the use of E14.5 because this stage offers the opportunity of analysing cells in different stages of differentiation. Accordingly, they interpret the difference across the proximo-distal axis as a reflection of different stages of RPE maturation. However, the vT and dT regions might also reflect properties related to the ciliary margins, which have a rather different specification program. Does the atlas reflect what currently known? Can the authors at least discuss this point?

Minor points
The use of PE vs RPE to define the less differentiated and differentiated epithelium is somewhat counterintuitive. The RPE acquire pigment as it differentiates so PE does not seem to identify the undifferentiated tissue. It may be easier to refer to undifferentiated vs differentiated RPE, for example uRPE vs RPE? Or prospective vs differentiated pRPE vs RPE.

At end of the first paragraph of the results: …., Smarcc2 expression was higher in the inner nuclear layer….. Should not be the RGC layer?

Reviewer 3

Advance summary and potential significance to field

Ovadia et al reports spatial transcriptomic profiling of the retinal pigment epithelium (RPE) and associated ocular mesenchyme in the E14.5 eye. Spatial data are obtained using laser-capture microdissection at 4 positions along the proximal-distal axis dorsally and ventrally (so 8 samples per tissue). The authors then use several computational approaches to segregate the data based on tissue, expression and putative gene regulatory networks enriched in the various tissue regions.

Comments for the author

Overall, the profiling aspect of the study, while descriptive, provides a very nice atlas for the developing RPE and mesenchyme. A limit is that data are solely from one time point, but E14.5 is a good choice given the activity and developmental differences along the P/D and D/V axes in these tissues at that time. As these approaches go, GEO-Seq is already a bit outdated given the advances in single-cell spatial barcoding and the use of Hi-plex RNA imaging strategies but, again, the data seem good and will be useful to the field. That being said, I do think some discussion of the strategy and strengths/weaknesses is warranted given these more recent technological advances in this type of experiment. Adding some biology, the authors focus on Swi/Snf activity and generate a conditional knockout of Smarcc1 and 2, which results in defects in the RPE. They then utilize GEO-Seq on these samples and identify some potential differences in RPE regulatory network activity and progenitor cell behaviors.

This aspect of the study is interesting, but a bit more speculative, with computational data only bolstered with a few expression analyses suggesting differences in expression at the transcriptomic and protein level for various factors. I'd have liked to see a bit more here linking some of the phenotypes back to the regulatory networks and thus, validating the process, beyond a few antibody stains.

Overall, I am positive about the manuscript and the contribution it makes to the field (in its current state) but would like to perhaps see a bit more depth in the functional analyses that bring the paper together and validate the model (and techniques) utilized. The paper is fine without this, it just leaves me as a reader and reviewer, wanting more.

First revision

Author response to reviewers' comments

We are grateful for the positive evaluation and the constructive comments of the three reviewers.

© 2023. Published by The Company of Biologists under the terms of the Creative Commons Attribution License (https://creativecommons.org/licenses/by/4.0/).

We have addressed the comments and introduced the required changes, as explained in our point-by-point responses below.

Reviewer #1:

1. Abstract, Introduction and Results (including Figure 1): ***It is too preliminary to comment on the tumour suppressive activity of these members of the SWI/SNF complexes based upon current assessment of proliferation and cell cycle using Ki67 immunostaining and application of the R package Seurat, respectively, as well as the thickening of the PE layer as demonstrated in Figure 1A. Similarly, on page 3, paragraph 1, line 6, the reference to tumor-suppressive role should be downplayed based upon the evidence presented.***

Response: We have changed the abstract from “The findings illustrate the tumor suppressive activity of the SWI/SNF complexes, and their role in controlling the intersection between RPE and neural cell fates in the developing mammalian eye”

to

“The findings illustrate the roles of the SWI/SNF complexes in controlling the intersection between RPE and neural cell fates and the coupling of cell-cycle exit and differentiation.”

We have also deleted “In agreement with their tumor-suppressive role” from introduction.

2. For Figure 1A, the differences in the thicknesses of the peripheral and central PE and NBL (neuroblastic layers) should be quantified. Additional assessment of proliferation and cell cycle progression could be accomplished through use of markers of S-phase (BrdU incorporation 30-45 minutes prior to embryo extraction) and M-phase (Phosphohistone H3 by immunofluorescence or immunohistochemistry). Alternatively, quantification of cell-cycle phases could be assessed by use of flow cytometry.

Response: We thank the reviewer for this comment. The pigmented progenitors are scarce and, as shown here, heterogeneous, thus preventing the use of flow cytometry to determine their cell-cycle stage. To determine the changes in cell proliferation in vivo, in the FcKO PE, we quantified the percentage of cycling cells (detection of Ki67 by antibody) or cells in the S phase (by EdU pulse labeling) in the four regions that correspond to the spatial transcriptomic analyses (Methods, New Fig. 5D–G). In the control, supporting the central-to-peripheral progression of RPE maturation, we detected a significantly higher percentage of proliferating cells (EdU+ and Ki67+) in the distal PE regions (CT, T) as compared to the proximal regions (CO, O). In the FcKO PE, these regional differences in cell proliferation were reduced and instead, we detected a significant increase in both EdU+ and Ki67+ cells in all spatial positions along the proximal– distal axis of the optic cup (Fig. 5E, G). These results support a key role for the SWI/SNF complexes in the inhibition of cell proliferation in the PE. This inhibition is evident in all regions, but is more pronounced in the more advanced stages of PE differentiation (O, CO).

The above findings are now incorporated into the results (pp. 10-11). Quantification of the significant increase in cell number in the FcKO PE is presented in Fig. S3.

3. Furthermore, it is unclear why apoptosis wasn't assessed either by use of expression of activated Caspase-3 in tissue sections or calculation of the sub-G1 peak using flow cytometry. How well does Seurat correlate with methods such as flow cytometry?

For Figure 6, are any of the observed differences between controls and full cKO statistically significant?

Response: We did not detect an increase in the number of cleaved caspase 3-positive (ccasp3+) cells in the FcKO PE compared to the control (N = 3; new Fig. S3 shows representative sections from control and FcKO PE labeled with ccasp3).

For the neuronal markers, we now add staining for N-Cad (CDH2), TUBB3 (TUJI), MAP2 and NF165 (NEFM) using specific antibodies (Figs. 6, 7N,R). We did not detect staining for any of these in the

© 2023. Published by The Company of Biologists under the terms of the Creative Commons Attribution License (https://creativecommons.org/licenses/by/4.0/).

control PE, but a marked elevation in transcripts (Fig. 6A) and protein for CDH2 in all regions and for TUBB3, MAP2 and NF165 in the O region in the FcKO PE. This was repeated in three eyes from three different embryos. The results are described in the revised version on pp. 11.

B. Specific concerns:

1. Methods

The authors should provide a rationale for use of the DCT-driver and also spell out dopachrome tautomerase for its first use.

Response: We generated PE-specific deletion of both *Smarcc1* and *Smarcc2* using the respective conditional alleles and the dopachrome tautomerase (DCT/Tyrp2) *DCT-Cre* driver, which is active in the pigmented progenitors of the optic cup (Choi et al., 2012; Davis et al., 2009; Tuoc et al., 2013b).

2. Please explain the opposite expression gradients of Pax6 and Sox9 for the reader.
Response: We have added the following to the Introduction: "In this regard, the TFs PAX6 and SOX9 have been documented to control the early and late stages, respectively, of RPE differentiation; *Pax6* is expressed transiently in the RPE progenitors, controlling the level and activity of *Mitf* and inhibiting the expression of *Sox9*, which in turn is expressed later in development and is required for the expression of RPE maturation genes such as *Rpe65, Vmd2, Anglp4* and *Vegf* (Cohen-Tayar et al., 2018; Raviv et al., 2014)."

3. How were specific regulons selected for more detailed presentation of the data and further discussion? Could others been instructive as well, such as Pole3 or Fli1?

Response: We highlighted representative regulons for which we identified enrichment for biological and cellular functions (presented in Fig. 3C) and for which there is published information on TF activity. To easily access the list of the regulons and associated genes for further evaluation, the interested reader can refer to Tables S5 and new S6 for controls, and Tables S10 and new S11 for FcKO.

4. Isl1 is not retinal ganglion cell specific (page 12, paragraph 1, line 6) although this is correctly explained in the discussion.

Response: This has been corrected: "the ganglion cell TFs POU4F2/BRN3b, or ganglion, amacrine and bipolar cell marker ISL1 (Gan et al., 1996; Martin-Partido and Francisco-Morcillo, 2015)(Fig S4E-H)."

3. Discussion

Please provide a reference delineating the various collagen genes expressed in Bruch's membrane.

Response: We have added two references describing the proteins considered to constitute the RPE and choroid basement membrane (Bai et al., 2009; Booij et al., 2010a; Booij et al., 2010b).

C. Minor Concerns

a. Page 2, paragraph 1, line 3: Add "an" prior to "important". Line 13: Add "stage" after "vesicle". DONE

b. Paragraph 2, line 13: Delete "the" prior to "RPE". DONE

c. Page 5, paragraph 1 line 8: add ",respectively" after "Me". DONE

d. Page 7, paragraph 2, line 1: Change "transcription" to "transcriptional". DONE

© 2023. Published by The Company of Biologists under the terms of the Creative Commons Attribution License (https://creativecommons.org/licenses/by/4.0/).

e. Page 10, paragraph 3, line 5: Replace "indicating" with "supporting". DONE

f. Page 15, paragraph 3, line 7: Delete "the" prior to "SWI/SNF". DONE

g. Page 16, paragraph 2, line 7: Add "(ganglion)" after Brn3b since this marker is specific for RGC. DONE

h. Page 17, paragraph 3, line 3: What is meant by "good morphology sections"?

Response: This part of the sentence has been deleted.

Reviewer #2:

1. It is somewhat surprising that Wnt signalling components are found enriched in C8, a Me cluster. This contrast with the notion that Wnt signalling is implicated in RPE specification (e.g, PMID: 19553286; 19596317). In the discussion in contrast, there is mention of Wnt signalling activation in the more proximal RPE regions. The data related to Wnt signalling should be better explained in the results to avoid confusions and considering the early role of this pathway in the activation of several RPE specific TFs.

Response: The detection of WNT-pathway genes in the peripheral ocular Me (the list of identified genes is given in Table S9) agrees with previous studies reporting the expression of WNT-pathway genes and the pathway's activity in the periocular mesenchyme (POM). To clarify this in the Results, we have added (p. 7): "The detection of WNT-pathway genes in the peripheral Me is in accordance with previous reports on the activity of this pathway in regulating periocular Me differentiation (Carpenter et al., 2015; Kumar and Duester, 2010). This adds to (supports?) the documented role of the canonical Wnt pathway in RPE differentiation (Fujimura et al., 2009; Westenskow et al., 2009)."

2. The authors indicate that cells of the FcKO RPE "transdifferentiated" or de- differentiate. These terms do not seem appropriate: the PE does not acquire RPE characteristic and therefore cannot transdifferentiate or de-differentiate. Quite likely, it simply retains neural progenitor characteristics failing to activate those required for RPE.

Response: DCT is an enzyme involved in pigmented biogenesis and is activated during RPE specification. Thus, the DCT-Cre is active after onset of specification. Note that we do detect MITF transcript and protein in the FcKO RPE (although MITF targets are not expressed). This supports activation of the RPE differentiation program prior to the induction of the conditional deletions. Thus, PE was specified and then partly transdifferentiated/dedifferentiated into aberrant neuronal progenitors.

To clarify and avoid confusing terminology, we changed the term "these aberrant SWI/SNF-deficient neural progenitors initiated the expression of early neural precursor genes" (p. 16).

3. The data related to the identity of the FcKO RPE is somewhat confusing. The F-C7 cluster contains genes belonging to synaptic transmission and synaptic organization, thus suggesting a rather differentiated state. However, the authors suggest the coexistence of a proliferative marker (Ki67) with an axonal protein (TuJ1) and the absence of retinal identity. Can the authors confirm and better document that Ki67+ cells are also TuJ1+? Tuj1 is considered a post-mitotic marker and it is possible that the FcKO RPE contains cells at different stages of specification/differentiation.

Response: To clarify this, we have included in new Fig. 6A, a heat map that presents the expression of several proliferation, neuronal progenitor and neural precursor genes that show elevation of their transcripts in the O and CO region of the FcKO PE We have also validated this by antibody labeling for some of the genes that showed elevated expression: CDH2 (N-cadherin) protein was detected throughout the FcKO RPE and we observed coexpression of MAP2 and TUBB3 in the Ki67+ cells (new Fig. 6). These additional data further support the failure to couple neuronal precursor gene expression with cell-cycle exit in at least some of the FcKO cells.

4. The lack of Vsx2 expression does not completely rule out the lack of retinal identity. Vsx2

© 2023. Published by The Company of Biologists under the terms of the Creative Commons Attribution License (https://creativecommons.org/licenses/by/4.0/).

and Mitf have been shown to regulate each other expression and the lack of Vsx2 might be somewhat related to an altered cross talk. Are other retinal enriched genes expressed in the FcKO RPE (e.g. Six6, Rx et,c)?

Response: Both *Rax* and *Six6* are among the differentially downregulated genes in the FcKO RPE. We have added the respective football plots for these genes (Fig. S4A,B; Table S7).

5. The authors have compared dorsal vs ventral RPE cells, reaching the conclusion that there are little differences or at least less than those observed along the proximo-distal axis. This is somewhat in contrast with the notion that the eye acquires a clear dorso-ventral polarity. It might be interesting to at least discuss this point.

Response: The differentially expressed genes between the dorsal and ventral regions are detailed in the Results (Fig. 2). The list of differentially expressed genes is presented in Table S2. It is beyond the scope of the current study to elaborate on the functional significance of the differentially expressed genes between dorsal and ventral positions, but the availability of the data will be valuable for future studies.

6. The authors justified the use of E14.5 because this stage offers the opportunity of analysing cells in different stages of differentiation. Accordingly, they interpret the difference across the proximo-distal axis as a reflection of different stages of RPE maturation. However, the vT and dT regions might also reflect properties related to the ciliary margins, which have a rather different specification program. Does the atlas reflect what currently known? Can the authors at least discuss this point?

Response: The data are available for further analysis based on the interest of the researchers using our webtool (https://shaiovadia.shinyapps.io/NewShiny_Publication/). To uncover the factors involved in the development and differentiation of the pigmented ciliary margin cells would require analyzing additional stages, along with validation and eventually functional studies. This is beyond the scope of this study, which primarily focused on RPE differentiation and the roles of the SWI/SNF complexes in regulating the RPE differentiation program. Note that the regions selected for analysis were predefined based on fixed distances from the tip and thus do not necessarily represent only those progenitors of the iris and ciliary body pigmented epithelia. Focusing on this population would require specifically isolating the relevant cells based on anatomical features and also probe additional stages to gain insight into the developmental trajectory. This is an interesting topic for research that we have planned for future studies.

7. The use of PE vs RPE to define the less differentiated and differentiated epithelium is somewhat counterintuitive. The RPE acquire pigment as it differentiates so PE does not seem to identify the undifferentiated tissue. It may be easier to refer to undifferentiated vs differentiated RPE, for example uRPE vs RPE? Or prospective vs differentiated pRPE vs RPE.

Response: We explained in the Introduction (p. 1) that “

The optic cups contain an outer layer of pigmented epithelium (PE) progenitors and an inner layer of retinal progenitor cells (RPCs). Most of the PE progenitors differentiate to form the RPE, while those located in the distal tip of the optic cup form the PE layers of the ciliary body and iris (Davis- Silberman and Ashery-Padan, 2008).” The term “PE” therefore refers to both RPE and the pigmented ciliary/iris progenitors.

8. At end of the first paragraph of the results: …., Smarcc2 expression was higher in the inner nuclear layer….. Should not be the RGC layer?

Response: To clarify, we have added: “SMARCC2 (BAF170) expression was higher in the inner nuclear layer, populated by ganglion and early-born amacrine precursors, as compared to low expression in the RPCs and PE (Fig. 1D, Fig. S1A).”

© 2023. Published by The Company of Biologists under the terms of the Creative Commons Attribution License (https://creativecommons.org/licenses/by/4.0/).

Reviewer #3:

1. Overall, the profiling aspect of the study, while descriptive, provides a very nice atlas for the developing RPE and mesenchyme. A limit is that data are solely from one time point, but E14.5 is a good choice given the activity and developmental differences along the P/D and D/V axes in these tissues at that time. As these approaches go, GEO-Seq is already a bit outdated given the advances in single-cell spatial barcoding and the use of Hi-plex RNA imaging strategies but, again, the data seem good and will be useful to the field. That being said, I do think some discussion of the strategy and strengths/weaknesses is warranted given these more recent technological advances in this type of experiment.

Response: We have added the following to the Discussion "in contrast to single-cell technologies where sequencing depth is low and spatial resolution is inferred based on clustering analyses"; also in the discussion "The transcriptomic data, which preserve the spatial coordinates, provide a basis for future studies implementing single-cell technologies, which will allow attributing positional information to the identified cell populations."

2. Adding some biology, the authors focus on Swi/Snf activity and generate a conditional knockout of Smarcc1 and 2, which results in defects in the RPE. They then utilize GEO-Seq on these samples and identify some potential differences in RPE regulatory network activity and progenitor cell behaviors.

This aspect of the study is interesting, but a bit more speculative, with computational data only bolstered with a few expression analyses suggesting differences in expression at the transcriptomic and protein level for various factors. I'd have liked to see a bit more here linking some of the phenotypes back to the regulatory networks and thus, validating the process, beyond a few antibody stains.

Response: In the revised manuscript, we have added a quantitative analysis of the cell cycle (by EdU pulse chase followed by quantification, revised Fig 5) as well as co-labeling of several neuronal precursor markers with Ki67 (Fig. 6). The findings support a key role for SWI/SNF in regulating both cell-cycle exit and the gene-regulatory networks of the RPE, as well as in inhibiting competing neuronal genes. These are substantial novel findings on important epigenetic layers of regulation in tissue maturation and differentiation.

3. Overall, the profiling aspect of the study, while descriptive, provides a very nice atlas for the developing RPE and mesenchyme. A limit is that data are solely from one time point, but E14.5 is a good choice given the activity and developmental differences along the P/D and D/V axes in these tissues at that time. As these approaches go, GEO-Seq is already a bit outdated given the advances in single-cell spatial barcoding and the use of Hi-plex RNA imaging strategies but, again, the data seem good and will be useful to the field. That being said, I do think some discussion of the strategy and strengths/weaknesses is warranted given these more recent technological advances in this type of experiment. Adding some biology, the authors focus on Swi/Snf activity and generate a conditional knockout of Smarcc1 and 2, which results in defects in the RPE. They then utilize GEO-Seq on these samples and identify some potential differences in RPE regulatory network activity and progenitor cell behaviors.

This aspect of the study is interesting, but a bit more speculative, with computational data only bolstered with a few expression analyses suggesting differences in expression at the transcriptomic and protein level for various factors. I'd have liked to see a bit more here linking some of the phenotypes back to the regulatory networks and thus, validating the process, beyond a few antibody stains.

Response: We thank the reviewer for appreciating the high-resolution spatial transcriptomic atlas of the developing RPE and adjacent ocular mesenchyme in control and SWI/SNF-deficient RPE. The detailed analysis of a single stage already revealed the major transcription factors and their gene-regulatory networks that function during the differentiation of RPE and adjacent ocular mesenchyme. The study further shows that the RPE-specific gene regulatory network is dependent on the activity of the SWI/SNF complexes, which are required for the expression and activity of RPE transcription factors and, at the same time, for the inhibition of the neural progenitor and cell-

© 2023. Published by The Company of Biologists under the terms of the Creative Commons Attribution License (https://creativecommons.org/licenses/by/4.0/).

proliferation genes (Fig. 8). The results place SWI/SNF at the intersection between the neural progenitor stage and the subsequent differentiation into the highly specialized eye lineages, both essential for normal retinal function. We have added, as a first paragraph to the Discussion, an explanation of the importance and novelty of the findings. We hope that the revised manuscript will be deemed acceptable for publication in Development.

Second decision letter

MS ID#: DEVELOP/2022/201488

MS TITLE: The SWI/SNF complexes are required for retinal pigmented epithelium differentiation and the inhibition of cell proliferation and neural differentiation programs

AUTHORS: Shai Ovadia, Guizhong Cui, Ran Elkon, Mazal Gulkar-Cohen, Nitay Zuk-Bar, Tran Touc, Naihe Jing, and Ruth Ashery Padan
ARTICLE TYPE: Research Article

I am delighted to tell you that your manuscript has been accepted for publication in Development, pending our standard ethics checks.

Reviewer 1

Advance summary and potential significance to field

The manuscript provide a new resource for the developmental biology community, a spatial transcriptomic atlas of the developing retinal pigmented epithelium (RPE) and adjacent ocular mesenchyme, using Geo-seq, a platform for geographical position sequencing. Although the work presented is focused one one mouse developmental stage E14.5, there is currently no other resource available, and the selected developmental stage includes a range/spectrum of progenitors and their respective differentiation programs. Moreover, using appropriate conditional inactivation strategies, the investigators have demonstrated an important role for the SWI/SNF chromatin remodeling complexes and their interaction with key transcription factors, in regulating RPE differentiation coordinated with concurrent neural progenitor differentiation programs.

Comments for the author

The authors have substantively addressed the concerns raised through the initial peer review of the manuscript. The revised version is significantly improved. No further revisions are requested.

Reviewer 2

Advance summary and potential significance to field

The manuscript has been revised addressing all the concerns raised. It will be an interesting addition to the field

Comments for the author

The manuscript has been revised addressing all the concerns raised. It will be an interesting addition to the field

© 2023. Published by The Company of Biologists under the terms of the Creative Commons Attribution License (https://creativecommons.org/licenses/by/4.0/).
